# High-Throughput Sequencing of Islet-Infiltrating Memory CD4^+^ T Cells Reveals a Similar Pattern of TCR Vβ Usage in Prediabetic and Diabetic NOD Mice

**DOI:** 10.1371/journal.pone.0076546

**Published:** 2013-10-17

**Authors:** Idania Marrero, David E. Hamm, Joanna D. Davies

**Affiliations:** 1 Torrey Pines Institute for Molecular Studies, San Diego, California, United States of America; 2 Adaptive Biotechnologies Corp, Seattle, Washington, United States of America; La Jolla Institute for Allergy and Immunology, United States of America

## Abstract

Autoreactive memory CD4^+^ T cells play a critical role in the development of type 1 diabetes, but it is not yet known how the clonotypic composition and TCRβ repertoire of the memory CD4^+^ T cell compartment changes during the transition from prediabetes to diabetes. In this study, we used high-throughput sequencing to analyze the TCRβ repertoire of sorted islet-infiltrating memory CD4^+^CD44^high^ T cells in 10-week-old prediabetic and recently diabetic NOD mice. We show that most clonotypes of islet-infiltrating CD4^+^CD44^high^ T cells were rare, but high-frequency clonotypes were significantly more common in diabetic than in prediabetic mice. Moreover, although the CD4^+^CD44^high^ TCRβ repertoires were highly diverse at both stages of disease development, dominant use of TRBV1 (Vβ2), TRBV13-3 (Vβ8.1), and TRBV19 (Vβ6) was evident in both prediabetic and diabetic mice. Our findings strongly suggest that therapeutic targeting of cells specifically expressing the dominant TCRβ might reduce pancreatic infiltration in prediabetic mice and attenuate the progression to diabetes.

## Introduction

Type 1 diabetes (T1D) is an autoimmune disease characterized by selective destruction of insulin-producing β cells within pancreatic islets. CD4^+^ and CD8^+^ T cells play essential roles in the initiation of β-cell destruction and disease progression [1], [2]. Nonobese diabetic (NOD) mice spontaneously develop T1D, and the disease shares many characteristics with human T1D [3]. T cells generally begin to infiltrate the islets at ∼3–4 weeks of age and continue to accumulate until β-cell destruction is sufficient to induce overt diabetes at about 12 weeks of age or later [4], [5].

Recent studies have shown that autoreactive CD4^+^ T cells with a memory phenotype can be isolated from the blood of diabetic patients and at-risk subjects [6]. In addition, islet-specific memory CD4^+^ T cells can be detected in the peripheral blood of both newly diagnosed patients and patients with clinical disease of several years duration [7], [8]. Islet-specific memory CD4^+^ T cells have also been implicated in the recurrent T-cell mediated autoimmune response after β-cell replacement [9], [10]. Studies examining the T cell receptor (TCR) β repertoire of memory CD4^+^ T cells in human T1D have been limited mostly to the analysis of peripheral T cells with antigen-specific tetramers. Using GAD65-specific tetramers, Laughlin et al. demonstrated that memory CD4^+^ T cell clones in peripheral blood of two T1D patients with recurrent autoimmunity express identical TCR Vβ and complementarity-determining region 3 (CDR3) sequences [7]. In NOD mice, Vβ, Dβ, and Jβ gene usage of islet-infiltrating antigen-specific CD4^+^ T cells has suggested significant skewing of Vβ gene usage [11]. Dominant TCR Vβ usage by effector/memory CD4^+^ T cells has also been shown in islet grafts and pancreases of diabetic NOD mice [12]. However, there have been no comparisons of the TCRβ repertoire and clonotypic composition of islet-infiltrating memory CD4^+^CD44^high^ T cells in prediabetic and diabetic NOD mice. The purpose of the current study was to identify the TCRβ repertoire of memory CD4^+^ T cells that spontaneously infiltrate the islets of diabetic NOD mice, and to compare that with the repertoire in prediabetic mice. We hypothesized that the TCRβ repertoires of islet-infiltrating memory CD4^+^ T cells in prediabetic and diabetic mice would overlap, but that some of the overlapping clonotypes would be significantly expanded in the diabetic mice.

Recognition of antigenic peptide/major histocompatibility complexes is mediated mainly by the third hypervariable complementary-determining region within the TCR β chain (CDR3β) [13]. The diversity of the TCR repertoire results from the variability of the CDR3β, which lies at the intersection between the Vβ, Dβ, and Jβ gene segments and contains random additions and deletions of noncoded N nucleotides at the V-D and D-J junctions. Thus, even when T cell clones express the same Vβ and Jβ genes, they can be distinguished by the unique combination of their CDR3 sequences (TCR clonotypes) [14].

In this study, we characterized the TCRβ repertoire of islet-infiltrating memory CD4^+^ T cells from prediabetic and diabetic NOD mice. High-throughput sequencing analysis of CD4^+^CD44^high^ cells from islets of individual mice showed a diverse repertoire composed mainly of rare clonotypes. High-frequency clonotypes were found in both prediabetic and diabetic mice but were more frequent within islets of diabetic mice. Unexpectedly, we found that islet-infiltrating CD4^+^CD44^high^ cells in prediabetic and diabetic mice showed comparable frequencies of TCRVβ gene usage dominated by TCR β-chain variable (TRBV)1 (Vβ2), TRBV13-3 (Vβ8.1), and TRBV19 (Vβ6). These gene segments were also predominant in the expanded clonotypes in diabetic mice. Our results reveal a high degree of TCRβ diversity among islet-infiltrating CD4^+^CD44^high^ cells of NOD mice and demonstrate that TRBV1, 13-3, and 19 gene segments are dominantly used within the memory repertoire in both prediabetic and diabetic mice.

## Materials and Methods

### Ethics Statement

Animal protocols were approved by the Institutional Animal Care and Use Committee (IACUC) at Torrey Pines Institute for Molecular Studies (TPI-10-07). Animal studies were performed in compliance with the institutional guidelines for animal care.

### Mice

Female NOD/ShiLtJ (NOD) mice were purchased from The Jackson Laboratory (Bar Harbor, ME). Mice were bred and housed in a specific pathogen-free animal facility at the Torrey Pines Institute for Molecular Studies (San Diego, CA).

### Diabetes Onset

Blood glucose levels (BGL) were measured weekly using Accu-Check Compact Plus (Roche Diagnostics, Indianapolis, IN). Mice with BGL >250 mg/dl were tested the next day, and diabetes onset was defined as two consecutive readings of >250 mg/dl. Mice were used at 10 weeks of age or within two days of diabetes onset. For the mice used in this study, diabetes onset occurred between 12 and 26 weeks of age. In our colony, 80% of female NOD mice develop diabetes by 28 weeks of age.

### Islet Isolation

Islets were isolated by collagenase perfusion of the main bile duct as previously described [15]. Briefly, the pancreas was exposed and perfused through the main bile duct with 3 ml of 2 mg/ml collagenase type V (Sigma-Aldrich, St. Louis, MO) prepared in Hank’s balanced salt solution (HBSS; Thermo Scientific* HyClone, Logan, Utah). The pancreas was then removed and incubated in ice-cold collagenase solution for 20 min at 37°C to digest the exocrine tissue. The digested tissue was dispersed by gentle shaking and digestion was stopped by the addition of cold HBSS supplemented with 10% fetal bovine serum (FBS; BioWhittaker™, Lonza, Walkersville, MD) followed by two washes with HBSS. The islets were purified by centrifugation on a discontinuous Ficoll PM 400 gradient (Sigma-Aldrich). The islets were collected at the interface between the 23.5% and 20% (w/v) layers.

### Isolation of Islet-infiltrating Cells

Purified islets were resuspended in RPMI 1640 medium (Thermo Scientific* HyClone) supplemented with 2 mM L-glutamine, 100 U/ml penicillin, 100 µg/ml streptomycin (all from Corning Cellgro, Mediatech Inc., Manassas, VA), 10 mM HEPES, 0.07% (w/v) sodium bicarbonate (BioWhittaker™), 1 mM sodium pyruvate (Gibco®, Life Technologies, Carlsbad, CA), 10% FBS, and 4 ng/ml recombinant murine interleukin-2 (PeproTech, Rocky Hill, NJ). Islets were incubated overnight in this medium at 37°C in a humidified 5% CO_2_ atmosphere. The next day, the islets and cell suspension were harvested and passed through a 40-µm strainer (BD Biosciences, San Jose, CA). The infiltrating cell suspension were collected and stained for cell sorting.

### Sorting of CD4^+^CD44^high^ Memory T cells

Islet-infiltrating cells were resuspended in sorting buffer (0.1% BSA in phosphate-buffered saline [PBS]), treated with Fc block (rat anti-mouse CD16/CD32) (BD Biosciences) and then stained with anti-CD4-APC and anti-CD44-PE monoclonal antibodies (mAbs) (BD Biosciences) on ice for 30 min. Cells were washed twice and resuspended at 5×10^6^/ml in 500 µl of sorting buffer. CD4^+^CD44^high^ cells were sorted on a FACSVantage flow cytometer with Diva software (BD Biosciences) at The Scripps Research Institute Flow Cytometry Core Facility (La Jolla, CA). Sorted cells were washed once in PBS and the cell pellet was frozen on dry ice for RNA extraction. The staining profile and gating strategy for cell sorting is shown in Figure S1.

### RNA Extraction and cDNA Synthesis

Total RNA was extracted from sorted CD4^+^CD44^high^ cells using the RNeasy Micro Kit (Qiagen, Valencia, CA). The entire sample was subjected to cDNA synthesis using the SuperScript™ First-Strand Synthesis System for RT-PCR (Life Technologies). The cDNA was frozen at −80°C until used for sequencing.

### High-throughput T cell Receptor Sequencing

TCRβ CDR3 regions were amplified and sequenced by Adaptive Biotechnologies Corp. (Seattle, WA). Briefly, a multiplex PCR system was designed to amplify all possible rearranged TCRβ CDR3 sequences from cDNA samples, using 35 forward primers specific for all Vβ gene segments and 13 reverse primers specific for all Jβ gene segments. The forward and reverse primers contain at their 5′ ends the universal forward and reverse primer sequences, respectively, compatible with the Illumina HiSeq cluster station solid-phase PCR system. The HiSeq system generates 60 base-pair (bp) reads, which cover the entire CDR3 lengths, sequencing from the J to the V region. The amplification and sequencing protocols have been described previously [16], [17]. The raw HiSeq sequence data were preprocessed to remove errors and to compress the data. Analysis of TCRβ sequences was conducted with the Adaptive TCR ImmunoSEQ assay. The TCRβ CDR3 region was defined according to the International ImMunoGeneTics (IMGT) collaboration [18], beginning with the second conserved cysteine encoded by the 3′ portion of the Vβ gene segment and ending with the conserved phenylalanine encoded by the 5′ portion of the Jβ gene segment. The number of nucleotides between these codons determines the length of the CDR3. A standard algorithm was used to identify which V, D, and J segments contributed to each TCRβ CDR3 sequence [18]. The protocol was validated for the samples collected here by Adaptive Biotechnologies. To determine reproducibility, sequencing of rearranged TCRβ CDR3 molecules was repeated for several RNA samples derived from low cell numbers, and the sequence copy numbers were compared on log scatter plots (Fig. S2). To determine PCR bias, each Vβ primer was PCR amplified and sequenced against all Jβ primers, and the combined data set for all V gene sequence numbers were recorded (Fig. S3). There is good assignment for the majority of V gene segments, except for two genes in the same family (12-1 and 12-2).

### Distribution of Frequencies and Cut-off Value

The raw frequencies of TCRβ CDR3 sequences were normalized by arithmetic mapping using a scale from 0 to 1, where 0 and 1 represent the lowest and highest frequencies, respectively. The normalization formula was:
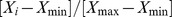
where *Χ_i_* is the raw frequency for an individual clonotype in an individual mouse, *Χ_min_* and *X_max_* are the lowest and highest raw frequencies, respectively [19].

A cut-off value to distinguish high and low-frequency clonotypes was calculated using the Z-score formula with a 99% confidence interval [20]:
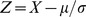
where *X* is the experimental value, *µ* is the mean value of all normalized frequencies from prediabetic mice, and *σ* is the number of standard deviations from the mean value.

### Statistical Analysis

Data were expressed as means ± standard error of the mean (SEM). Differences between two groups were compared using either the Mann-Whitney test or the unpaired two-tailed t test. Statistical significance is shown as *, p<0.05; **, p<0.005, ***, p<0.0005, and ****, p<0.0001. Significance was determined using the data presented in each figure. Statistical analyses were conducted using GraphPad Prism software.

## Results

### High-frequency Clonotypes are more Common in Islet-infiltrating CD4^+^CD44^high^ Cells in Diabetic than in Prediabetic NOD Mice

We analyzed the TCRβ repertoire of sorted islet-infiltrating CD4^+^CD44^high^ cells from individual NOD mice at 10 weeks of age (prediabetic; n = 7) and within 2 days of T1D onset (diabetic; n = 9). The multiplex PCR-based method amplifies the complete CDR3 region of all rearranged TCRβ genes. As such, we were able to generate a picture of the spontaneously emerging CD4^+^CD44^high^ TCRβ repertoire in individual NOD mice.

A total of 354,302 CDR3β sequences were obtained from islet-infiltrating CD4^+^CD44^high^ cells isolated from seven prediabetic mice. From these, 15,923 unique clonotypes (distinct CDR3β sequences) were identified. In contrast, only 1,471 unique clonotypes were identified from the 66,767 total CDR3β sequences obtained from nine diabetic mice (Table 1). Approximately tenfold more unique clonotypes were assembled from prediabetic than from diabetic mice, which can be attributed to the ∼tenfold higher number of CD4^+^CD44^high^ cells present in and sorted from the pancreases of the prediabetic mice. Indeed, the mean number of sorted CD4^+^CD44^high^ cells per mouse was 6,434±4,443 cells (range 1,320 to 13,538) for prediabetic mice and 669±636 cells (range 94 to 1,987) for diabetic mice.

**Table 1:** CDR3β sequences of islet-infiltrating CD4^+^CD44^high^ T cells.

	NOD mouse #	Total CDR3β sequences^1^	Total unique clonotypes^2^	Entropy^3^
**Prediabetic**	1	39,521	1,989	0.441
	2	7,147	388	0.724
	3	42,349	798	0.717
	4	46,142	7,692	0.635
	5	6,141	2,049	0.984
	6	193,763	2,822	0.896
	7	19,241	185	0.846
**Total**	n = 7	354,304	15,923	
**Diabetic**	8	555	26	0.440
	9	2,468	849	0.983
	10	9,634	80	0.291
	11	1,506	139	0.673
	12	1,120	37	0.462
	13	12,990	90	0.819
	14	4,470	86	0.690
	15	627	70	0.780
	16	33,397	94	0.444
**Total**	n = 9	66,767	1,471	

1 Total number of TCRβ CDR3 sequences amplified.

2 Total number of unique clonotypes identified.

3 Shannon’s entropy was used to quantify diversity of the TCRβ repertoire. Higher values indicate greater diversity.

**Figure 1:**
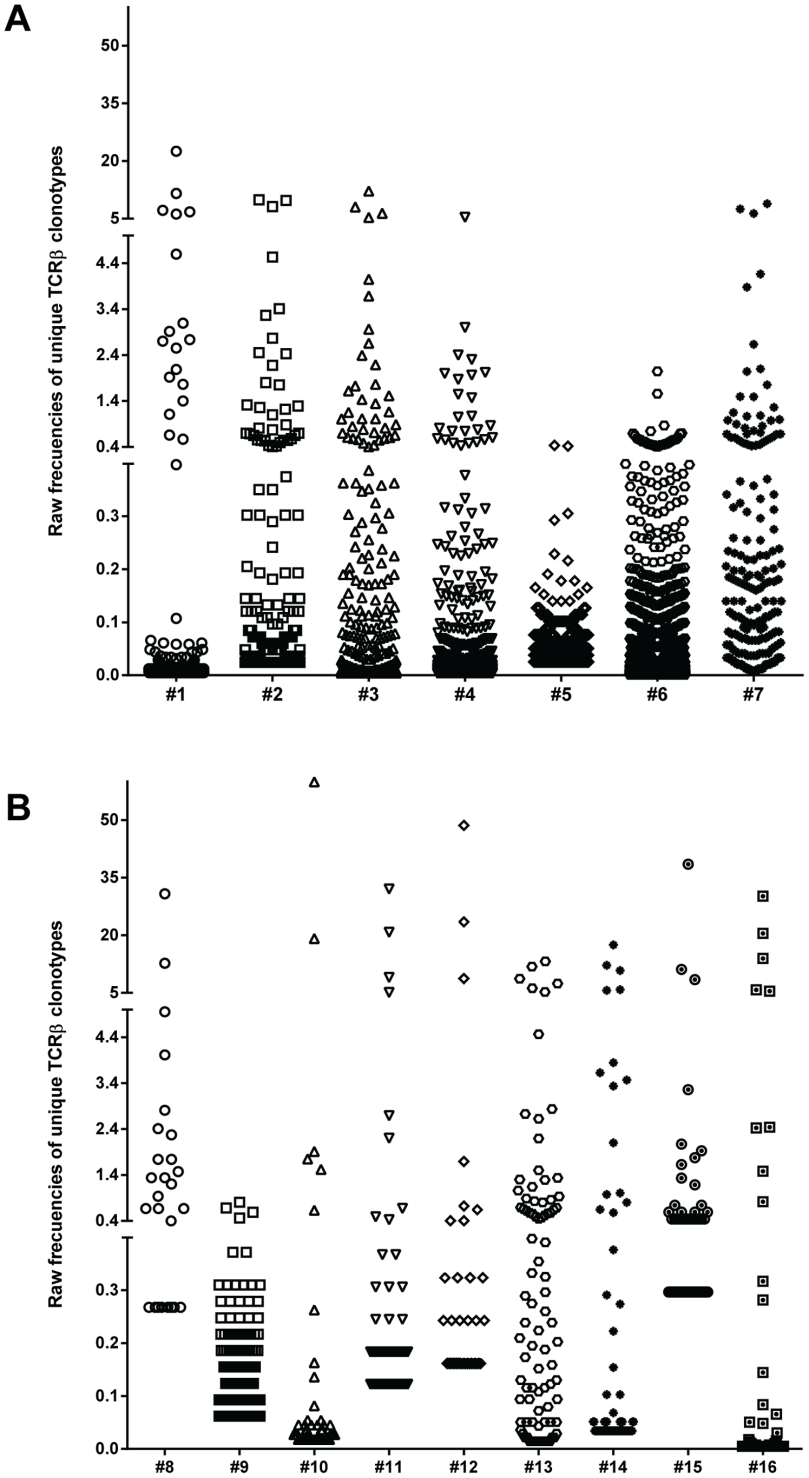
Distribution of unique CD4^+^CD44^high^ clonotypes in prediabetic and diabetic NOD mice. The frequency of individual TCRβ clonotypes was determined in CD4^+^CD44^high^ cells sorted from the islets of 10-week-old prediabetic (n = 7) and newly diabetic (n = 9) NOD female mice. Distribution of raw (non-normalized) frequencies among the unique TCRβ clonotypes in the memory repertoire is shown for each prediabetic (A) and diabetic (B) mouse. Each unique TCRβ clonotype is shown as a dot. The number of unique TCRβ clonotypes for each mouse is shown in Table 1.

The relative frequency of each clonotype assembled from CD4^+^CD44^high^ cells sorted from individual mice is shown in Figure 1 (raw data before normalization). The data show that in both prediabetic and diabetic mice, the islet-infiltrating memory cell repertoires are complex, consisting of a few high-frequency clonotypes and a large number of low-frequency clonotypes. Such complexity might indicate the involvement of different clonotypes at distinct stages of the autoimmune response. As mentioned above, the number of isolated cells, and therefore unique CDR3β sequences assembled, was generally lower for the diabetic than prediabetic mice. Therefore, the raw frequency of each clonotype for each mouse was normalized as described in the Materials and Methods section.

**Figure 2:**
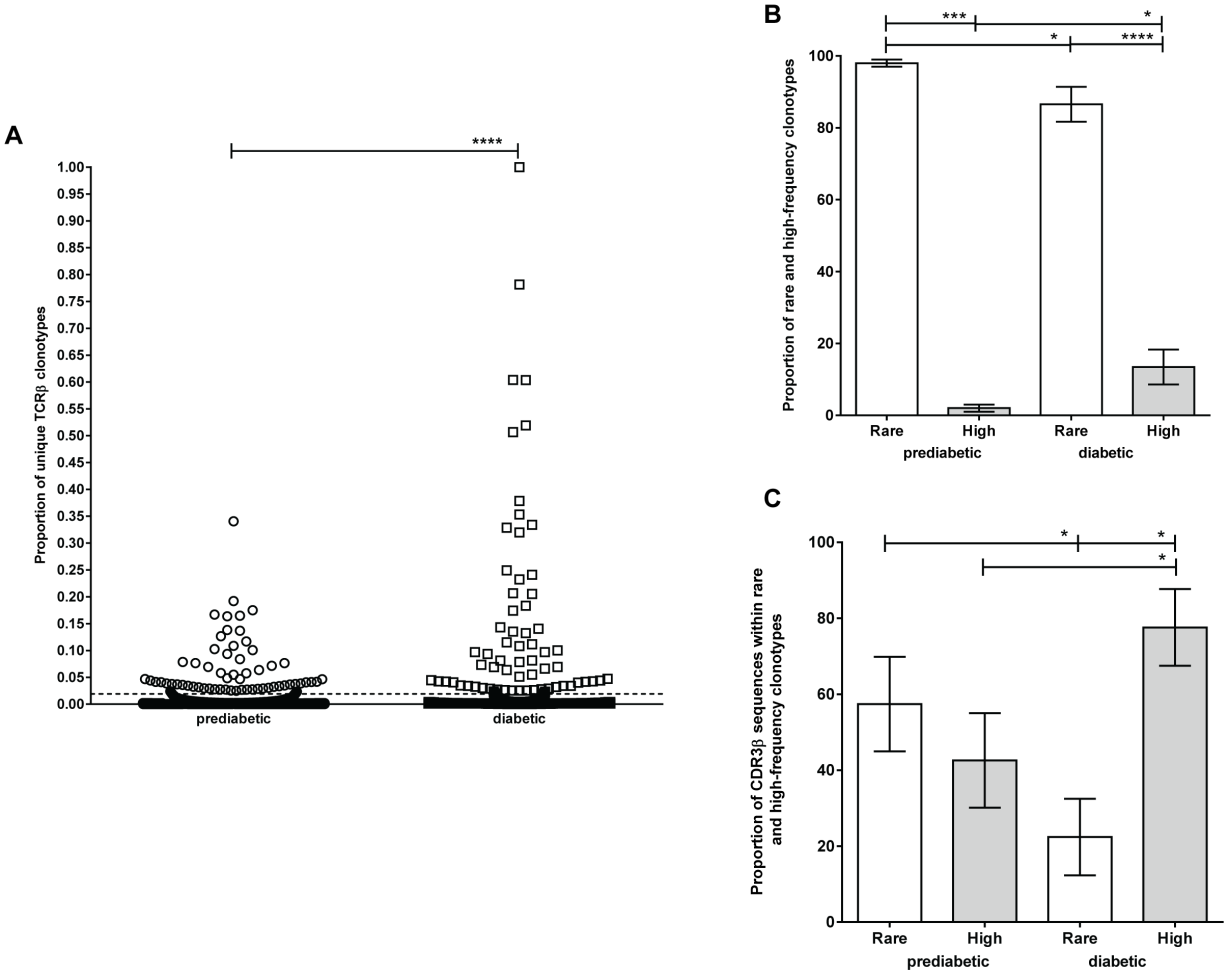
High-frequency CD4^+^CD44^high^ clonotypes are more common in the islets of diabetic than prediabetic NOD mice. CD4^+^CD44^high^ cells from the islets of 10-week-old prediabetic (n = 7) and newly diabetic (n = 9) NOD female mice were sorted and subjected to high-throughput sequencing of the CDR3β regions. (A) Scatter plot showing the normalized frequencies of all CDR3β sequences represented by unique TCRβ clonotypes (15,923 in prediabetic mice and 1,471 in diabetic mice, shown as dots). Clonotypes accounting for <0.02% and ≥0.02% of all CDR3βs were defined as rare and high-frequency clones, respectively. (B) The proportion of rare (white bar) and high-frequency (gray bar) clonotypes in prediabetic and diabetic mice. Rare clonotypes are significantly more common than high-frequency clonotypes in prediabetic (p = 0.0006) and diabetic mice (p<0.0001). Rare clonotypes are significantly more frequent in prediabetic than in diabetic mice (p = 0.0118), and high-frequency clonotypes are significantly more frequent in diabetic than in prediabetic mice (p = 0.0118). (C) The proportion of CDR3β sequences derived from rare (white bar) and high-frequency (gray bar) clonotypes in prediabetic or diabetic mice. The proportion of CDR3β sequences derived from rare clonotypes is significantly higher in prediabetic than in diabetic mice (p = 0.0168). The proportion of CDR3β sequences derived from high-frequency clonotypes is significantly higher in diabetic than in prediabetic mice (p = 0.0168). The proportion of CDR3β sequences derived from high-frequency clonotypes is significantly higher than sequences derived from rare clonotypes in diabetic mice (p = 0.0399).

A scatter plot comparing the distribution of normalized frequencies of all unique clonotypes between prediabetic and diabetic mice is shown in Figure 2A. Consistent with previous reports [21], [22] and with our non-normalized data (Fig. 1), we found that most of the memory clonotypes from both groups of mice are of low frequency. However, the memory TCRβ repertoire from diabetic mice contained significantly more high-frequency clonotypes than prediabetic mice (p<0.0001).

To distinguish between rare and high-frequency clonotypes, we calculated a cut-off value (see Materials and Methods) from the mean of the normalized frequencies of all unique clonotypes from prediabetic mice, considering that prediabetic mice would contain fewer expanded clones. From this calculation, unique clonotypes with normalized frequencies of <0.02% or ≥0.02% of total CDR3β sequences were defined as rare (low frequency) or high frequency, respectively. Specifically, 15,851 and 1,401 rare clonotypes were found in prediabetic and diabetic mice, respectively (Fig. 2A). Rare clonotypes constituted a significantly higher proportion of the total clonotypes in prediabetic than in diabetic mice (97.98% [range 92.97–100%, n = 7] vs. 86.55% [range 50–100%, n = 9], p = 0.0118) (Fig. 2B). Correspondingly, high-frequency clonotypes represented a significantly higher proportion of total clonotypes in diabetic than in prediabetic mice (13.45% [range 0–50%] vs. 2.02% [range 0–7.03%], p = 0.0118) (Fig. 2B). Although prediabetic and diabetic mice contained approximately the same number of high-frequency clonotypes (72 and 70, respectively), these high-frequency clonotypes accounted for a higher proportion of total CDR3β sequences in the diabetic mice than in the prediabetic mice (77.61% [range 0–97.27%] vs. 42.59% [range 0–83.07%, p = 0.0168) (Fig. 2C). In turn, a significantly smaller proportion of CDR3β sequences were represented by rare clonotypes in the diabetic mice than in prediabetic mice (22.39% [range 2.73–100%] vs. 57.41% [range 16.93–100%, p = 0.0168) (Fig. 2C).

These data demonstrate a clear change in the proportion of rare and high-frequency CD4^+^CD44^high^ clonotypes in the islets during the transition from prediabetes to diabetes. This could be due to a loss of rare clonotypes and/or to an expansion of high-frequency clonotypes in the islets of diabetic mice.

To quantify the diversity of CDR3β clones among the islet-infiltrating CD4^+^CD44^high^ cells in prediabetic and diabetic mice, Shannon’s entropy was calculated [23]. Shannon’s entropy is defined as the uncertainty in predicting the sequence identity of an individual sequence randomly selected from the dataset. For comparison, entropy was normalized by the number of unique productive sequences. This produces a metric that varies between 0 and 1, where 1 is the normalized entropy value if every sequence is at the same frequency in the population, and 0 is the entropy value if every sequence is derived from a single clone. We found that the entropy values were not significantly different (prediabetic mice 0.75±0.2 vs. diabetic mice 0.62±0.2), suggesting that the frequency distribution of CD4^+^CD44^high^ cells isolated from the prediabetic and diabetic mice is similar when the entire repertoire is considered (Table 1).

Our dataset of islet-infiltrating memory CD4^+^ TCRβ CDR3 sequences is available online at https://main.g2.bx.psu.edu/u/islet-memory/h/islet-memory-cd4tcells at the Galaxy website [24]–[26].

### The Same Dominant TCR Vβ Genes are Expressed by Islet-infiltrating Memory CD4^+^CD44^high^ Cells in Prediabetic and Diabetic Mice

**Figure 3:**
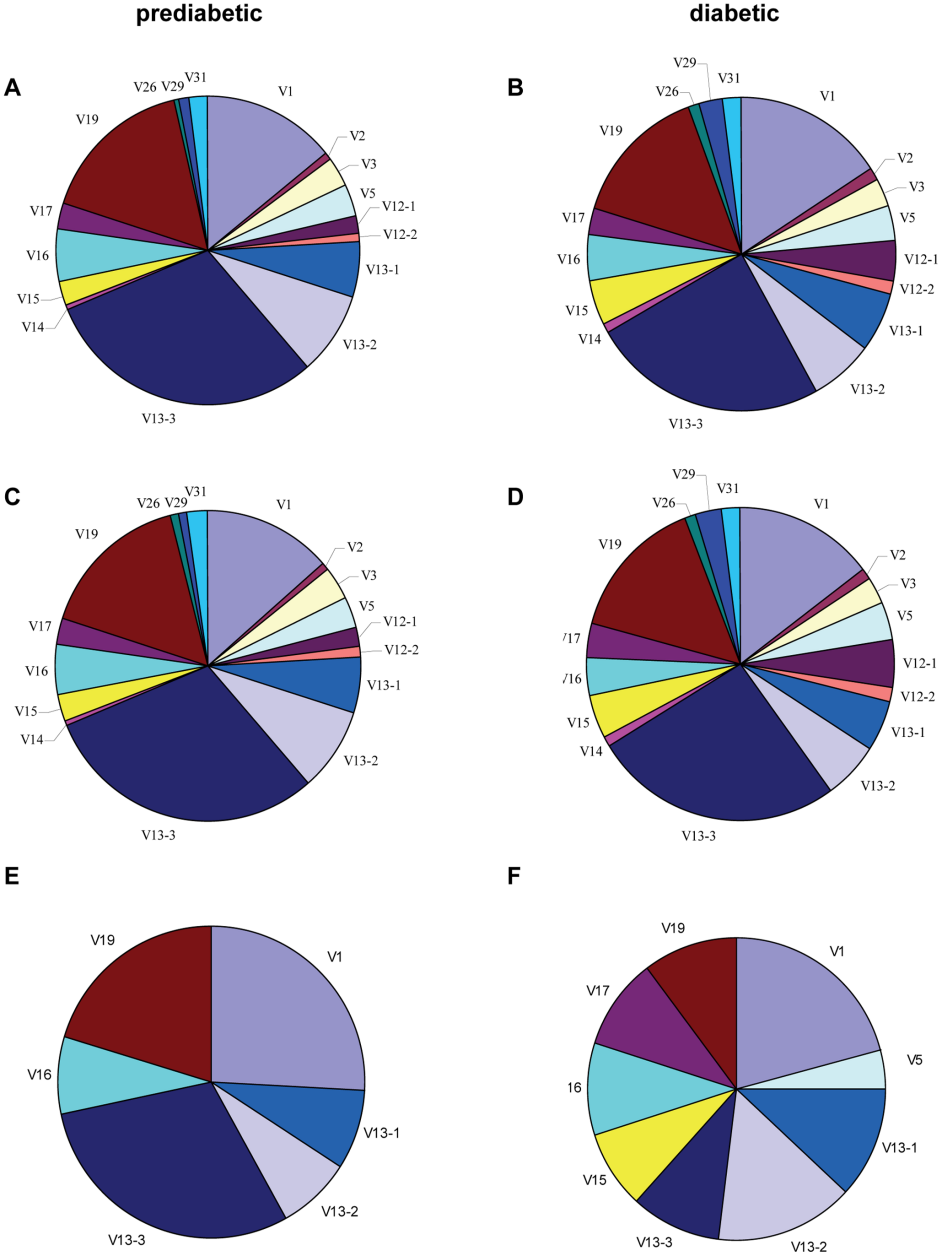
Islet-infiltrating CD4^+^CD44^high^ cells from prediabetic and diabetic mice exhibit the same dominant TRBV gene restriction. Islet-infiltrating CD4^+^CD44^high^ cells were sorted from prediabetic (A, C, E) and diabetic (B, D, F) mice and the CDR3β regions were sequenced. The average frequency of TRBV gene segment usage is shown for all clonotypes (A and B), rare clonotypes (C and D), and high-frequency clonotypes (E and F). Each TRBV gene segment is represented by a slice proportional to its frequency. TRBV4, 20, 21, 22, 23, 24, and 30 genes were used at very low frequencies and are not shown in the graphs.

To confirm the repertoire diversity and identify any disease-related changes in TCR Vβ gene usage by islet-infiltrating CD4^+^CD44^high^ cells, we next examined the frequency of TRBV gene segment usage by unique clonotypes in prediabetic and diabetic mice. When all clonotypes were analyzed, the usage frequencies of TRBV genes in the two CD4^+^CD44^high^ repertoires was remarkably similar, with the same three TRBV genes dominantly expressed in both groups. Thus, TRBV13-3 (Vβ8.1), TRBV19 (Vβ6), and TRBV1 (Vβ2) were expressed by 30.2%, 16.5%, and 14.2%, respectively, of all clonotypes from prediabetic mice (Fig. 3A) and by 26.9%, 16.1%, and 17.4% of all clonotypes from diabetic mice (Fig. 3B). Moreover, this was also observed when the mice were analyzed individually (Fig. S4). As expected, further analysis of the repertoires showed that TRBV usage by the rare clonotypes, including the dominantly restricted genes, was virtually identical to that of all clonotypes in both prediabetic and diabetic mice (Fig. 3). Thus, the percentage of rare clonotypes using TRBV13-3, TRBV19 and TRBV1 was 30.6%, 15.7%, and 13.5%, respectively, in prediabetic mice (Fig. 3C) and 30.8%, 15.8%, and 16.7% in diabetic mice (Fig. 3D).

High-frequency clonotypes in prediabetic mice showed a highly restricted TRBV usage dominated by TRBV13-3 (37.5%), TRBV19 (25.6%), and TRBV1 (32.5%) (Fig. 3E). In contrast, high-frequency clonotypes isolated from the diabetic mice showed predominant usage of TRBV1 (30.5%), TRBV13-2 (22.2%), and TRBV13-1 (17.2%) gene segments, although TRBV19 (15.1%) and TRBV13-3 (14.4%) were also expressed (Fig. 3F). Other TRBV gene segments were present in high-frequency clonotypes, but these were detected in only one diabetic and one prediabetic mouse. The frequency of TRBV gene segment usage by total, high-frequency, and rare clonotypes from individual mice is shown in Table S1.

Our data show that the CD4^+^CD44^high^ cells isolated from prediabetic and diabetic mice express an equally diverse TCRβ repertoire dominated by the same TRBV gene segments. These data suggest that the TCRβ repertoire of islet-infiltrating CD4^+^CD44^high^ cell is comparable in both groups of mice and that clonotypes using the dominant TRBV genes might be preferentially selected in the repertoire of islet-infiltrating cells in the NOD mice.

### TCR Jβ usage by Infiltrating CD4^+^CD44^high^ Cells is Highly Variable in Prediabetic and Diabetic Mice

**Figure 4:**
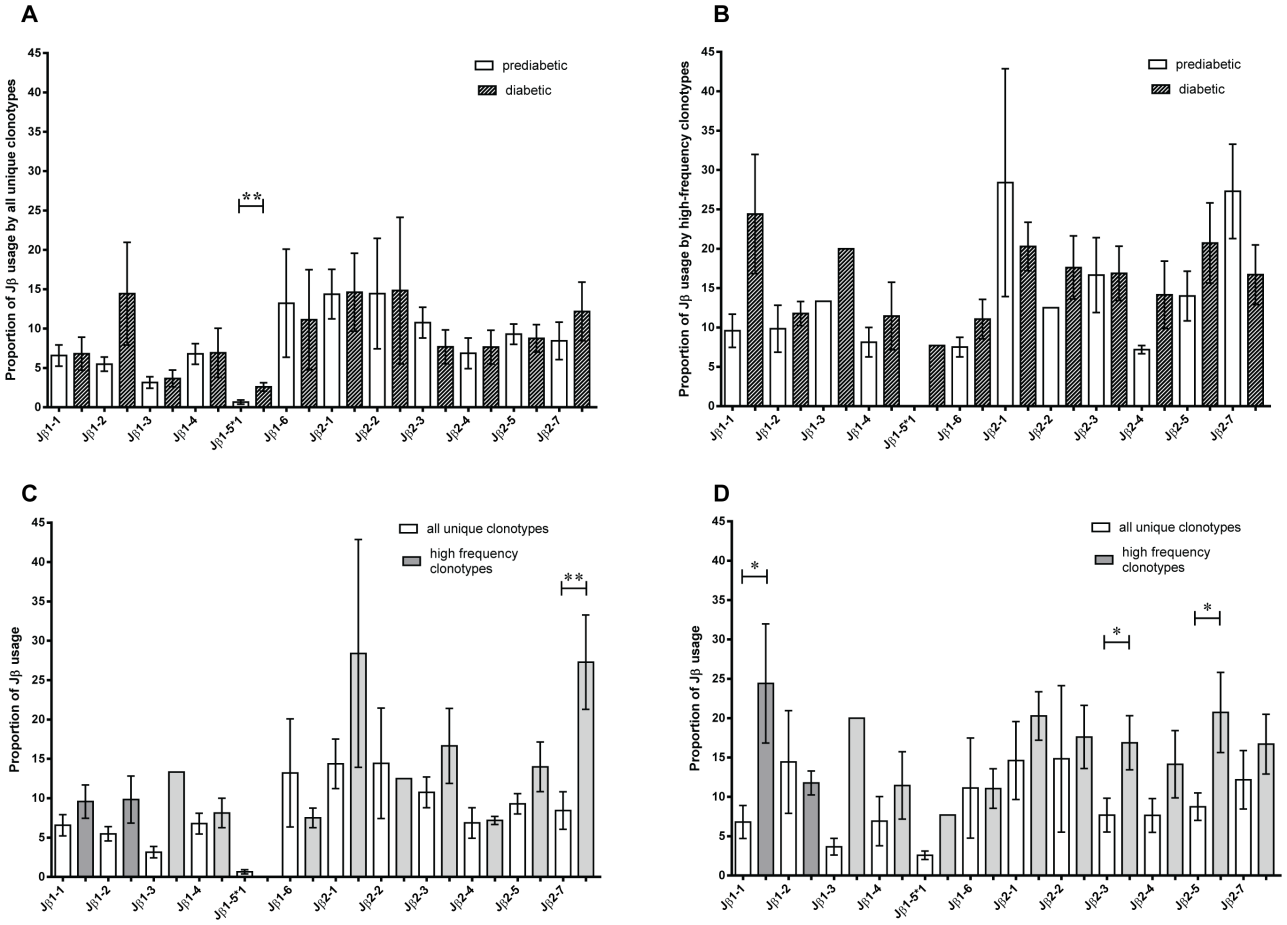
High variation in TRBJ usage in islet-infiltrating CD4^+^CD44^high^ T cell clonotypes. The average frequency of TRBJ gene usage by all CD4^+^CD44^high^ clonotypes (A) and high-frequency clonotypes (B) from prediabetic mice (**p = 0.0054 for TRBJ1-5*1) and diabetic mice. The average frequency of TRBJ gene usage by all CD4^+^CD44^high^ clonotypes and high-frequency clonotypes from (C) prediabetic mice (**p = 0.0081 for TRBJ2-7) and (D) diabetic mice (*p = 0.019 for TRBJ1-1, *p = 0.04 for TRBJ2-3, and *p = 0.022 for TRBJ2-5). All data are the mean ± SEM.

Because the similarity in the dominant TRBV gene usage of islet-infiltrating memory CD4^+^ T cells in prediabetic and diabetic mice was unexpected, we extended our analysis to examine TRBJ gene usage in these cells. Our data show that all known functional TRBJ genes were represented within the clonotypes from prediabetic and diabetic mice (Fig. 4A). The frequency of TRBJ usage within all clonotypes ranged from 14% for TRBJ2-1 and TRBJ2-2 to 0.7% for TRBJ1-5*1 in prediabetic mice, and from 14% for TRBJ1-2, TRBJ2-1, and TRBJ2-2 to 2.6% for TRBJ1-5*1 in diabetic mice (Fig. 4A). The only difference in TRBJ usage was found for TRBJ1-5*1, which was more common in diabetic mice (0.7±0.3% vs. 2.6±0.5%). TRBJ usage among rare clonotypes was the same as for all clonotypes, as expected (data not shown). Among the high-frequency clonotypes, TRBJ usage ranged from 28.4% for TRBJ2-1 to 7.2% for TRBJ2-4 in prediabetic mice and from 24.4% for TRBJ1-1 to 7.7% for TRBJ1-5*1 at diabetes onset (Fig. 4B). Although differences in gene usage were observed, these did not reach statistical significance, possibly due to large inter-individual variations. However, we found significant differences in TRBJ usage when the total and high-frequency clonotypes were compared. Thus, TRBJ2-7 was more common in high-frequency clonotypes in prediabetic mice (27.3±6.0% vs. 8.4±2.4%; Fig. 4C), whereas TRBJ1-1 was more common in high-frequency clonotypes in diabetic mice (24.4±7.6% vs. 6.8±2.1%; Fig. 4D). TRBV2-3 (16.9±3.4% vs. 7.7±2.1%) and TRBJ2-5 (20.7±5.1% vs. 8.7±1.7%) were also more common in high-frequency clonotypes than total clonotypes in diabetic mice (Fig. 4D).

Collectively, these data show that, although TRBJ gene usage was highly variable among islet-infiltrating CD4^+^ clonotypes at both stages of disease, the high-frequency clones in both groups of mice showed preferential usage of specific TRBJ genes, suggesting that clonotypes using these TRBJ segments might be under selective pressure to expand in response to islet antigens.

### CDR3β Length Distribution is Comparable in Islet-derived CD4^+^CD44^high^ Cells from Prediabetic and Diabetic Mice

**Figure 5:**
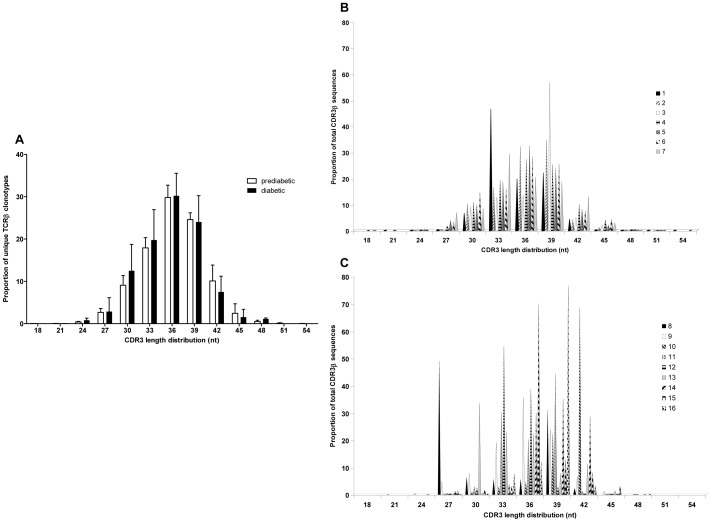
CD4^+^CD44^high^ clonotypes from prediabetic and diabetic mice have similar CDR3β length distribution. (A) The distribution of CDR3 lengths for all unique CD4^+^CD44^high^ clonotypes are presented as means ± SEM of prediabetic (white bar) and diabetic (black bar) mice. The distribution of CDR3β lengths are shown for individual prediabetic mice (B) or diabetic mice (C).

We next examined the CDR3β nucleotide (nt) length in the CD4^+^CD44^high^ clonotypes. We found no significant differences in the length distribution when all clonotypes were analyzed, with the mean length being 36 nt in both prediabetic (range 18–54 nt) and diabetic (range 24–48 nt) mice (Fig. 5A). When the individual prediabetic mice were examined, most of the mice showed similar CDR3 length distributions (Fig. 5B). In contrast, the individual diabetic mice showed markedly different patterns of CDR3β lengths (Fig. 5C). This finding might reflect the higher proportion of high-frequency clonotypes in the diabetic mice, as has previously been described for memory repertoires [27].

These results therefore indicate clonal expansion in the memory CD4^+^CD44^high^ repertoire of diabetic mice, as demonstrated by the skewed CDR3β length distribution in the high-frequency clonotypes of individual mice.

### Distinct Monoclonal Expansions Dominate the Islet-infiltrating CD4^+^CD44^high^ TCRβ Repertoire in Individual Diabetic Mice

**Figure 6:**
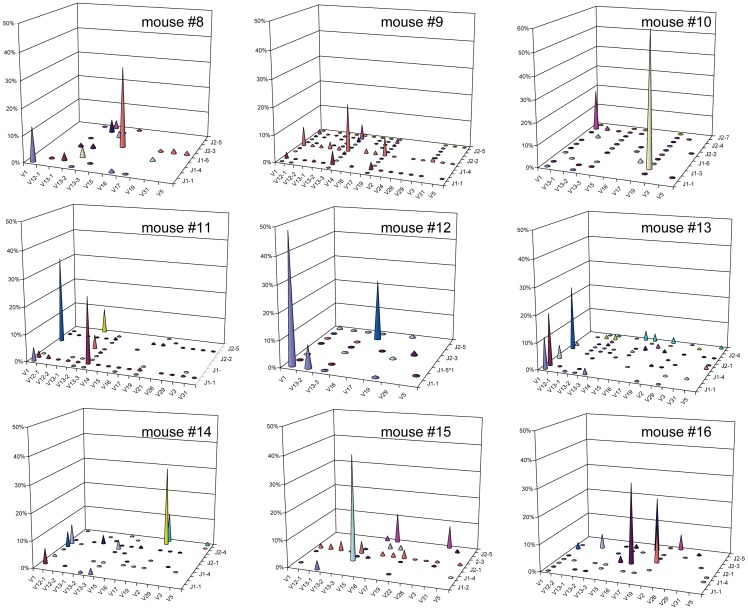
High-throughput sequencing analysis reveals monoclonal expansions in islet-infiltrating CD4^+^CD44^high^ cells from diabetic mice. The frequencies of V and J gene segment usage within total CDR3β sequences amplified from islet-infiltrating CD4^+^CD44^high^ cells isolated from individual diabetic. Each panel represents one mouse.

**Figure 7:**
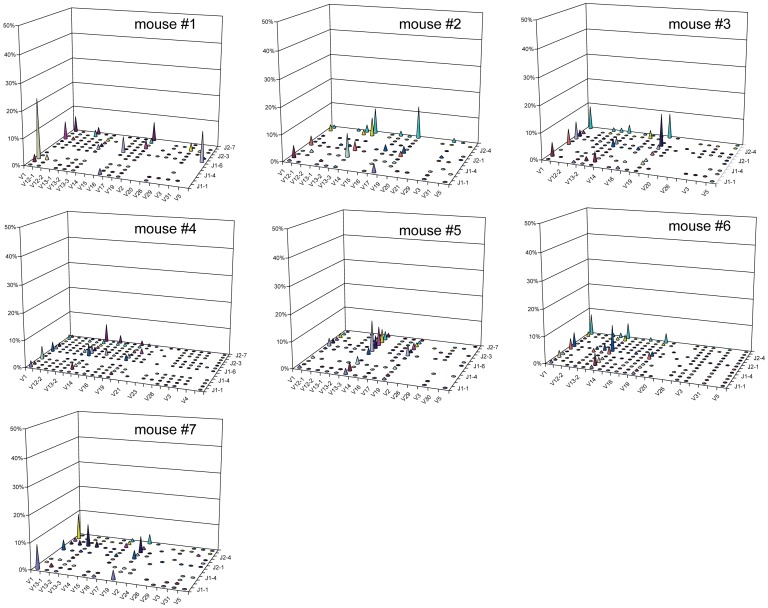
Absence of monoclonal expansions in prediabetic mice. The frequencies of V and J gene segment usage within total CDR3β sequences amplified from islet-infiltrating CD4^+^CD44^high^ cells isolated from individual prediabetic (B) mice. Each panel represents one mouse.

Our CDR3β sequencing results showed that high-frequency CD4^+^CD44^high^ clonotypes are more common in diabetic than in prediabetic mice (Fig. 2). We next analyzed monoclonal expansions within the high-frequency clonotypes; for this, we defined monoclonal expansions as clonotypes accounting for >15% of total CDR3β sequences in each mouse. Interestingly, we found that the repertoire of islet-infiltrating CD4^+^CD44^high^ cells in seven of the nine diabetic mice was dominated by monoclonal expansions (Fig. 6). Each mouse had one dominant monoclonal expansion, contributing between 22.93% (mouse #14) and 68.66% (mouse #10) of total CDR3β sequences, and a second smaller expansion (Fig. 6 and Table 2). In addition, one of the seven mice had a third monoclonal expansion. When the CDR3 sequences and gene segments were analyzed, we found that all of the monoclonal expansions were unique to each mouse. In addition, of the 14 monoclonal expansions, 10 expressed the dominant TRBV gene segments identified in the memory repertoire (four TRBV1, four TRBV19, and two TRBV13-3). In sharp contrast to our findings in diabetic mice, only one of the seven prediabetic mice (mouse #1) had a monoclonal expansion, which also used the dominant TRBV1 (Fig. 7).

**Table 2:** Monoclonal expansions dominate the islet-infiltrating CD4^+^CD44^high^ T cell repertoire at diabetes onset^1^.

Mouse^2^	TRBV gene	TRBJ gene	CDR3 sequence^3^	CDR3 Length^4^	Frequency^5^
**8**	15	2-1	SPGAEQFF	27	41.4
	1	1-1	SADNRVDTEVFF	39	17.1
**10**	19	1-3	STGTARSGNLYF	42	68.7
	1	2-5	SAGGQGLDTQYF	39	22.0
**11**	1	2-2	SASGQNTGQLYF	39	34.8
	13-3	1-2	SDPGGSDYTF	33	22.6
**12**	1	1-1	SAAQNTEVFF	33	53.7
	16	2-5	SFRDRKDTQYF	36	26.0
**14**	19	2-5	SAGTGGRQDTQYF	42	22.9
	19	2-5	RPWGNQDTQYF	36	15.9
**15**	13-3	1-6	SDRGTNSLYF	36	41.5
**16**	16	2-1	SLVGGNYAEQFF	39	35.6
	17	2-5	SREGVNQDTQYF	39	24.3
	19	2-2	SIWGGNTGQLYF	39	16.5

1 Monoclonal expansions are defined as clonotypes representing >15% of total CDR3β sequences.

2 Numbers correspond to the diabetic mice identified in Table 1. Only the seven mice having monoclonal expansions are shown.

3 Amino acid sequences between the conserved cysteine and phenylalanine residues, as defined by IMGT [57].

4 CDR3 length of nucleotides.

5 Raw frequency of each clonotype within the total CDR3β sequences obtained from each mouse.

Collectively, these results demonstrate that monoclonal expansions are common in the CD4^+^CD44^high^ repertoire of diabetic but not prediabetic mice. The diversity of the expanded clonotypes in the diabetic mice might be a reflection of the multiplicity of islet antigens associated with T1D.

### A Subset of Islet-infiltrating CD4^+^CD44^high^ Clonotypes is Present in both Diabetic and Prediabetic Mice

Because we observed evidence of monoclonal expansion in the CD4^+^CD44^high^ T cell repertoire of prediabetic as well as diabetic mice, we investigated whether clonotypes in the islet-infiltrating cells in diabetic mice might already be present at 10 weeks of age. Of the unique clonotypes present in individual prediabetic mice, we found a mean of 1.35% (range, 0.88–1.71%) were also present in the diabetic memory repertoire. Remarkably, 15.44% (range, 6.98–46.15%) of the unique clonotypes in the islets of individual diabetic mice were also present in the repertoire at 10 weeks of age, suggesting that that such clonotypes might be involved in diabetes development.

Of the 17,394 unique CD4^+^CD44^high^ clonotypes identified in the prediabetic (15,923) and diabetic mice (1,471), we found that 1,150 were common to more than one mouse. Of these, 148 were present in at least one prediabetic and one diabetic mouse, 993 were present in at least two prediabetic mice, and nine were present in at least two diabetic mice. Of the 148 clonotypes present in both groups of mice, 13 clonotypes were present in four or more mice (Table 3), suggesting that these clonotypes may be preferentially selected on MHC class II A^g7^. One clonotype, TRBV13-2-GSGTTNTEVFF-TRBJ1-1, was present in six of the seven prediabetic mice and three of the nine diabetic mice. Notably, this was a high-frequency clonotype in the diabetic but not the prediabetic mice, which strongly suggests that this clonotype may have undergone antigen-driven expansion between 10 weeks of age and diabetes onset. In addition, clonotypes TRBV1-STRGTEVFF-TRBJ1-1, TRBV13-3-SGTANSDYTF-TRBJ1-2, and TRBV15-SRDSSYEQYF-TRBJ2-7 were present in three prediabetic and two diabetic mice. Interestingly, we found that rare clonotypes were more likely to be present in multiple prediabetic mice than diabetic mice, suggesting that there is a selection process for these cells to either enter or survive in the pancreas. The remaining 135 clonotypes common to both groups of mice were shared by at least one mouse from each group (Table S2). Of the 993 CD4^+^CD44^high^ clonotypes found only in the prediabetic mice, 945 (95.2%) were rare clonotypes that were present in only two mice.

**Table 3:** Islet-infiltrating CD4^+^CD44^high^ cell clonotypes shared by prediabetic and diabetic NOD mice^1^.

			Prediabetic NOD mice						Diabetic NOD mice							
CDR3 sequence^2^	TRBV	TRBJ	#1	#2	#3	#4	#5	#6	#9	#10	#11	#12	#13	#14	#15	#16
STRGTEVFF	1	1-1			0.04^3^	2.0		0.12		0.63						0.14
SATANSDYTF	1	1-2			0.004	0.01			0.09				6.26			
SAGGNYAEQFF	1	2-1	0.004			0.01		0.002	0.31							
SDGTGEDTQYF	13-1	2-5	2.5			0.01	0.02			1.91						
GSGTTNTEVFF	13-2	1-1	0.04	0.05	0.03	0.11	0.3	0.004			0.49	8.73		0.65		
SGTANSDYTF	13-3	1-2			0.01	0.04		0.57	0.31	0.02						
SDRGANSDYTF	13-3	1-2	0.004		0.004	0.007	0.02	0.07		0.03						
SGDSSYNSLYF	13-3	1-6	0.004			0.003	0.04									0.005
SDAEQFF	13-3	2-1	0.007			0.005	0.06		0.09							
SDWGGYAEQFF	13-3	2-1	0.007			0.008	0.09		0.28							
SLGAGYAEQFF	15	2-1				0.61							0.05		0.74	2.44
SRDSSYEQYF	15	2-7	0.06			0.14		0.01					0.01		1.78	
SPGTANTEVFF	19	1-1	0.004			0.007	0.05				0.18					

1 Amino acid sequences of CDR3β shared by at least four mice.

2 Amino acid sequences between the conserved cysteine and phenylalanine residues, as defined by IMGT [57].

3 Raw frequency of clones with the indicated sequence (%).

Overall, these findings demonstrate that several CD4^+^CD44^high^ clonotypes present in the islets of diabetic mice are already present in prediabetic mice. These clonotypes might therefore represent persistent islet-infiltrating memory CD4^+^ T cells.

### Some TCRβ Clonotypes from Islet-infiltrating CD4^+^CD44^high^ Cells have Previously Been Reported in T1D

We predicted that the CD4^+^CD44^high^ clonotypes common to multiple mice may have previously been sequenced and reported in other studies. On comparing the CDR3β amino acid sequences of our unique clonotypes with published CDR3 sequences amplified from NOD mouse islets, we found that 12 of the CDR3β sequences using the same TRBV and TRBJ gene segment have been reported by others (Table 4, Ref [28]–[30]). Surprisingly, these sequences were not necessarily present in multiple mice. Six of the 12 sequences were isolated from islets of NOD mice between 8 and 12 weeks of age (Table 4A, Ref [28]–[30]).

**Table 4:** Comparison of published CDR3β sequences with CDR3β sequences from islet-infiltrating CD4^+^CD44^high^ T cells^1^.

	CDR3 sequence^2^	TRBV	TRBJ	Reference	CDR3 sequence^3^	TRBV	TRBJ	Mouse^4^	Frequency^5^
**A**	SDRNTEVFF	13-3	1-1	[28]	SDRNTEVFF	13-3	1-1	4	0.010
	SDRGRAEQFF	13-3	2-1	[28]	SDRGRAEQFF	13-3	2-1	4	0.003
	SGQDQDTQYF	13-3	2-5	[30]	SGQDQDTQYF	13-3	2-5	2	0.04
	SRDSAETLYF	19	2-3	[29]	SRDSAETLYF	19	2-3	4	0.006
	GGDSSYEQYF	13-2	2-7	[29]	GGDSSYEQYF	13-2	2-7	2	0.69
					GGDSSYEQYF	13-2	2-7	3	0.56
					GGDSSYEQYF	13-2	2-7	4	0.089
	SIDGGRAETLYF	19	2-3	[29]	SIDGGRAETLYF	19	2-3	3	12.21
					SIDGGRAETLYF	19	2-3	4	0.003
					SIDGGRAETLYF	19	2-3	10	0.018
**B**	SLNTEVFF	15	1-1	[11]	SLNTEVFF	15	1-1	7	0.016
	RPGGRDYAEQFF	15	2-1	[11]	RPGGRDYAEQFF	15	2-1	7	0.025
	SRDSSYEQYF	15	2-7	[11]	SRDSSYEQYF	15	2-7	1	0.06
					SRDSSYEQYF	15	2-7	4	0.086
					SRDSSYEQYF	15	2-7	4	0.065
					SRDSSYEQYF	15	2-7	6	0.01
					SRDSSYEQYF	15	2-7	13	0.01
					SRDSSYEQYF	15	2-7	15	1.78
	GGYEQYF	13-2	2-7	[31]	GGYEQYF	13-2	2-7	4	0.008
	SIRGTEVFF	19	1-1	[32]	SIRGTEVFF	19	1-1	5	0.025
	SDSQNTLYF	13-3	2.4	[27]	SDSQNTLYF	13-3	2.4	1	0.0048

1 CDR3 sequences between the conserved cysteine and phenylalanine residues, as defined by IMGT [57].

2 Published CDR3 sequences.

3 CDR3 sequences amplified from unique CD4^+^CD44^high^ clonotypes in this study.

4 Mouse numbers identified in Table 1.

5 Raw frequency of clonotypes with the indicated sequence (%).

The remaining six CDR3β sequences identified by us and others have been reported to bind antigenic epitopes relevant to T1D (Table 4B, Ref [11], [27], [31], [32]). Three were amplified from islet-infiltrating CD4^+^ T cells specific for the mimetic peptide recognized by the BDC2.5 clonotypic TCR [11]. Of the three remaining sequences, one was reported to be from an islet-specific clone 4-1-K.1 [31], another combines with the conserved TRAV5D-4*04 α chain of an insulin B:9-23 peptide-specific T cell hybridoma [32], and the third was identified as an immunodominant CDR3β in the CD8^+^ T cell response to IGRP_206–214_ [27].

We found 12 additional CDR3β sequences that were identical to published CDR3 sequences amplified from NOD mouse islets, each of which we found was associated with a different TRBV gene segment (Table 5). Three were isolated from islet infiltrates of prediabetic NOD mice (Table 5A, Ref [28], [30], [33]) and two were sequences isolated from islet-infiltrating CD4^+^ T cells from 14- to 18-day-old NOD mice [34]. The remaining seven CDR3β sequences have been reported to bind antigenic epitopes relevant to T1D, but we found the same CDR3 sequence combined with different TRBV gene segments (Table 5B, Ref [11], [31], [35]).

**Table 5:** Comparison of CDR3β sequences from CD4^+^CD44^high^ T cells and published sequences differing in TRBV usage^1^.

	CDR3 sequence^2^	TRBV	TRBJ	Reference	CDR3 sequence^3^	TRBV	TRBJ	Mouse^4^
**A**	SRDWGSQNTLYF	13	2-4	[28]	SRDWGSQNTLYF	17	2-4	1
	SGQDQDTQYF	14	2-5	[30]	SGQDQDTQYF	13-3	2-5	2
	RGTGGYAEQFF	29	2-1	[33]	RGTGGYAEQFF	16	2-1	9
	SRDGSAETLYF	5	2-3	[34]	SRDGSAETLYF	17	2-3	5
	SPDRDEQYF	5	2-7	[34]	SPDRDEQYF	19	2-7	4
**B**	RLGNQDTQYF	15	2-5	[31]	RLGNQDTQYF	13-3	2-5	4
	SLGGNQDTQF	15	2-5	[35]	SLGGNQDTQF	16	2-5	4
	SLNTEVFF	15	1-1	[11]	SLNTEVFF	19	1-1	5
					SLNTEVFF	19	1-1	1
	SLGGYAEQFF	15	2-1	[11]	SLGGYAEQFF	3	2-1	4
	SLGQQDTQYF	15	2-5	[11]	SLGQQDTQYF	12-1	2-5	4
					SLGQQDTQYF	16	2-5	6
	SPDRGQDTQYF	15	2-5	[11]	SPDRGQDTQYF	13-3	2-5	4
					SPDRGQDTQYF	13-3	2-5	4
	SLDNQDTQYF	15	2-5	[11]	SLDNQDTQYF	12-2	2-5	4

1 CDR3 sequences between the conserved cysteine in all TRBV genes and phenylalanine, as defined by IMGT [57].

2 Published CDR3 sequences.

3 CDR3 sequences amplified from unique CD4^+^CD44^high^ clonotypes in this study.

4 Mouse numbers identified in Table 1.

It is interesting that the same CDR3β amino acid sequence can result from rearrangement of different TRBV gene segments. This suggests that the recognition of similar islet-specific antigens by different TRBV gene segments might contribute to amplification of the autoimmune response in the pancreas.

Collectively, the data presented here indicate that islets of prediabetic mice contain CD4^+^CD44^high^ cells with CDR3β sequences known to be relevant to islet antigen specificity.

## Discussion

In this study, we analyzed the clonotypic composition and CDR3β diversity of sorted islet-infiltrating CD4^+^CD44^high^ T cells from individual prediabetic and diabetic NOD mice. We found that the repertoire of islet-infiltrating CD4^+^CD44^high^ cells in both groups of mice dominantly used TRBV1 (Vβ2), TRBV13-3 (Vβ8.1), and TRBV19 (Vβ6) gene segments. This finding provides a strong rationale for targeting a limited number of TCR elements as a therapeutic approach to preventing and treating autoimmune diabetes. Consistent with this, Liu et al. have shown that selective mAb-mediated depletion of Vβ13a^+^ T cells prevented diabetes onset in both the LEW.1WR1 rat model and the spontaneous BBDP rat model [36].

To our knowledge, the observation that islet-infiltrating CD4^+^CD44^high^ cells in NOD mice show preferential expression of TRBV1, TRBV13-3, and TRBV19 has not previously been reported. However, islet-infiltrating T cells from prediabetic NOD mice have been described to preferentially express Vβ6 (TRBV19), Vβ8.2 (TRBV13-2), Vβ1 (TRBV5), and Vβ7 (TRBV29) [29], [30], [33], [37]. Two other studies examined the TCRβ repertoire of individual NOD mice by conventional sequencing of PCR products amplified from single sorted islet-infiltrating CD4^+^ T cells or single islet-infiltrating sIA^g7^-mBDC^+^ CD4^+^ T cells. In these studies, restriction to TRBV15 (Vβ12) and TRBV5 (Vβ1) was observed, although the number of sequences examined was small [11], [34]. However, neither study reported the memory or naïve phenotype of the sequenced CD4^+^ T cells. In contrast, we found that approximately 3.5% and 3.6% of islet-infiltrating CD4^+^CD44^high^ cells expressed TRBV5 (Vβ1) and TRBV15 (Vβ12), respectively.

We have also examined TRBV usage by pancreatic lymph node CD4^+^ T cells in our mice (Marrero et al., unpublished results), and the relative frequencies of TRBV1 (6.3±1.2%), TRBV19 (9.1±1.0%), and TRBV13-3 (23±0.9%) are comparable to those reported by Barker et al. [34] in peripheral CD4^+^ T cells. However, we found that peripheral memory CD4^+^CD44^high^ cells show more frequent usage of TRBV1 and TRBV13-3 (19.5±6.8% and 28.3±5.4%, respectively), suggesting that these cells are expanded in the periphery before they migrate to the pancreas. In contrast, TRBV19 usage in peripheral CD4^+^CD44^high^ T cells (9.1±1.2%) was no different, suggesting that these cells might expand at a different location. In contrast to the islet-infiltrating cells, we found that CD4^+^CD44^high^ cells from pancreatic lymph nodes do not dominantly express TRBV1, TRBV13-3, and TRBV19 (Marrero et al., unpublished results).

A recent study using multiparameter flow cytometry identified restricted Vβ usage by effector/memory CD8^+^ and CD4^+^ T cells infiltrating the host pancreas and islet grafts in individual diabetic NOD mice [12]. In this study, Diz et al. found preferential use of Vβ8.1/2, Vβ8.3, Vβ6, Vβ12, and Vβ2 by pancreas-infiltrating memory CD4^+^ T cells, which is consistent with our data, with the exception of Vβ12. This apparent discrepancy might be due to several methodological differences between the two studies, including the memory CD4^+^ T cell subsets analyzed (total CD4^+^CD44^high^ cells in our study compared to effector/memory CD4^+^ cells in the study by Diz et al.), the method of isolation of pancreas-infiltrating CD4^+^ T cells, and the methodology used to analyze the TCRβ repertoire. We used a highly sensitive approach that sequenced the complete CDR3β region. It is possible that rare or low-frequency clones were underestimated or undetected using the multiparameter flow cytometry approach.

The predominance of rare clonotypes within the islet-infiltrating CD4^+^CD44^high^ compartment at both stages of disease was surprising. Although this has been observed in memory CD4^+^ and CD8^+^ T cell repertoires in PBMC of healthy human donors [16], [18], we are not aware that it has been reported in target tissue under autoimmune attack. One study showed that islet antigen-specific CD4^+^ T cells accumulate in the islets of NOD mice, but bystander CD4^+^ T cells are unable to enter and/or accumulate in the islets [38]. A more recent study showed that islet antigen-specific CD4^+^ T cells are the first to infiltrate the islets [39] and that non-islet-specific CD4^+^ T cells are recruited to the islets by the inflammatory response [40]. Here, we found that rare clonotypes are significantly more frequent in prediabetic mice than diabetic mice. In addition, some CDR3β sequences from our rare clonotypes have previously been associated with islet antigen reactivity, indicating that autoantigen-specific T cell clones can be present at a very low frequency in the memory CD4^+^ repertoire. However, we do not yet know if the rare clonotypes are the first to enter the pancreas, if they are predominantly islet-specific, or if they are capable of mediating β cell destruction [41].

Why large numbers of rare clonotypes dominate the prediabetic islet memory CD4^+^ repertoire is unclear. One possibility is that the rare clonotypes provide a diverse set of islet antigen-specific and/or nonspecific memory CD4^+^ T cells that are actively recruited to and retained within the pancreas in response to inflammation [39]. These cells might provide a sufficiently large T cell repertoire to ensure immune-mediated destruction of the target tissue, as one may expect during a pathogen-directed response. Although this is a new concept, it is consistent with the idea that an optimal T cell response to any antigen requires a diverse TCR repertoire [21], which might equally apply to responses to autoantigens. An alternative explanation for the predominance of rare clonotypes in the CD4^+^CD44^high^ repertoire is that they represent an influx of Tregs in response to the expansion of pathogenic effector cells [42], [43]. Indeed, high TCR diversity has been shown to be optimal for Treg-mediated suppression of experimental acute graft versus host disease [44]. The mechanism by which TCRβ diversity is generated within the pancreas of prediabetic NOD mice, and whether such TCRβ diversity may be beneficial or detrimental, remain to be determined.

Our study demonstrates that the islet-infiltrating CD4^+^CD44^high^ TCRβ repertoire is diverse in individual prediabetic and diabetic NOD mice. We found that the proportion of rare clonotypes was significantly lower in diabetic than in prediabetic mice. Moreover, significantly more CD4^+^CD44^high^ T cells were sorted from the pancreases of prediabetic than of diabetic mice, suggesting that rare CD4^+^CD44^high^ clonotypes are lost between 10 weeks of age and diabetes onset. Rare clonotypes may exit from or die within the pancreas, possibly as a result of competition with higher affinity clones that expand to become high-frequency clonotypes. We have previously shown that the TCR Vβ repertoire is more diverse in the pancreatic lymph nodes of 10-week-old NOD mice than in either 4-week-old or newly diabetic mice [45]. Together with the results presented here, these data suggest that 10 weeks of age might be an important checkpoint for ensuring maximal diversity of the islet-infiltrating CD4^+^CD44^high^ T cell repertoire.

TRBV1, TRBV13-3, and TRBV19 have been shown previously to be relevant to disease evolution in T1D [32], [46]–[50]. Bacelj et al. showed that administration of an anti-Vβ8 mAb prevented cyclophosphamide-induced diabetes in NOD/Wehi mice [51] and disease recurrence in pancreas isografts in diabetic NOD mice [52]. In addition, treatment with anti-Vβ8 mAb, but not anti-Vβ9 or anti-Vβ13 mAbs, prevented the development of hyperglycemia and insulitis after streptozotocin treatment of C57BL/KsJ mice [53]. Similarly, young NOD mice rapidly developed diabetes following transfer of clones expressing TRBV1, TRBV19, TRBV13-1, or TRBV15 with or without the B:9-23-specific Vα13 chain, demonstrating that pathogenicity was conferred by the Vβ and not the Vα chain [47]. Finally, diabetogenic CD4^+^ T cells depleted of Vβ6 (TRBV19) have a reduced capacity to transfer disease to young NOD mice [54]. In our study, most of the CD4^+^CD44^high^ monoclonal expansions identified in both the prediabetic and the diabetic mice expressed TRBV1, TRBV19, or TRBV13-3. These clonally expanded T cells might therefore represent cells expressing TCRs with high avidity for islet antigens [55] that are present early in disease development and that proliferate vigorously in the islets of diabetic NOD mice [56].

## Conclusions

To our knowledge, this is the first study to evaluate the clonotypic composition of islet-infiltrating CD4^+^CD44^high^ cells in the NOD mouse by high-throughput sequence analysis. Our results demonstrate that the TCRβ repertoire of these cells is diverse and consists mainly of rare clonotypes. Nevertheless, there is clear evidence of TRBV1, TRBV13-3, and TRBV19 restriction at both the prediabetic stage and at the onset of diabetes. We also show that many CD4^+^CD44^high^ clonotypes present in the islets of diabetic mice were already present in the prediabetic mice, suggesting that potentially diabetogenic clonotypes are resident in the pancreas before the onset of diabetes. Collectively, our study suggests that targeting cells expressing the dominant TRBV gene segments may have therapeutic benefit in NOD mice and humans at risk of diabetes and recurrent autoimmunity.

## Supporting Information

Figure S1 **Flow cytometric cell-sorting strategy for isolation of CD4^+^CD44^high^ cells.** Islet-infiltrating cells were co-stained with fluorescent antibodies specific for CD4 and CD44. The figure shows representative FACS profiles of one 10-week-old NOD mouse and the strategy used for sorting. Regions R1 (panel a), R2 (panel b), and R3 (panel c) indicate selection of lymphocytes and exclusion of doublets. Region R4 in panel d identifies CD4^+^ cells. Panel e shows the expression of CD44 on gated CD4^+^ T cells (R4). The marker in panel e shows the region used to sort memory CD4^+^CD44^high^ cells, which were defined as CD4^+^ T cells expressing the highest level of CD44 (in this case 29.8% of CD4^+^CD44^high^ cells).
(TIF)

Figure S2 **Validation of the sequencing protocol.** Sequence copy counts of TCRβ CDR3 regions obtained by sequencing the same cDNA sample from prediabetic mouse #1 (A) and diabetic mouse #14 (B) in two separate PCR reactions. Each point in the log–log scatter plot represents a unique clone. Points in green were found in both reactions and those in blue or yellow were found in one PCR reaction.
(TIF)

Figure S3 **PCR bias assessment.** Each Vβ primer was PCR amplified and sequenced against all Jβ primers. After processing, all TRBV gene sequences were mapped and recorded. The graph shows the combined data set for all TRBV genes. The bars correspond to the number of TRBV segments correctly assigned (white), the number miss-assigned to a particular TRBV gene (black) and the number of sequences incorrectly assigned from a particular TRBV gene (grey).
(TIF)

Figure S4 **TRBV gene usage by islet-infiltrating CD4^+^CD44^high^ clonotypes from individual NOD mice.** The frequency of TRBV gene usage in each of the prediabetic (A) and newly diabetic (B) mice.
(TIF)

Table S1 Frequency of TRBV gene segment usage by individual mice. The frequency of TRBV gene segment usage by total, high-frequency, and rare islet-infiltrating CD4^+^CD44^high^ clonotypes.
(DOC)

Table S2 Islet-infiltrating CD4^+^CD44^high^ clonotypes shared by at least one prediabetic and one diabetic mouse. 135 clonotypes common to both groups of mice were shared by at least one mouse from each group.
(DOC)
