# Supplementary figures and images for: High-Throughput Sequencing of Islet-Infiltrating Memory CD4+ T Cells Reveals a Similar Pattern of TCR Vβ Usage in Prediabetic and Diabetic NOD Mice
Source: PLoS One. 2013 Oct 17;8(10):e76546. doi: 10.1371/journal.pone.0076546 (PMC3798422; doi:10.1371/journal.pone.0076546)

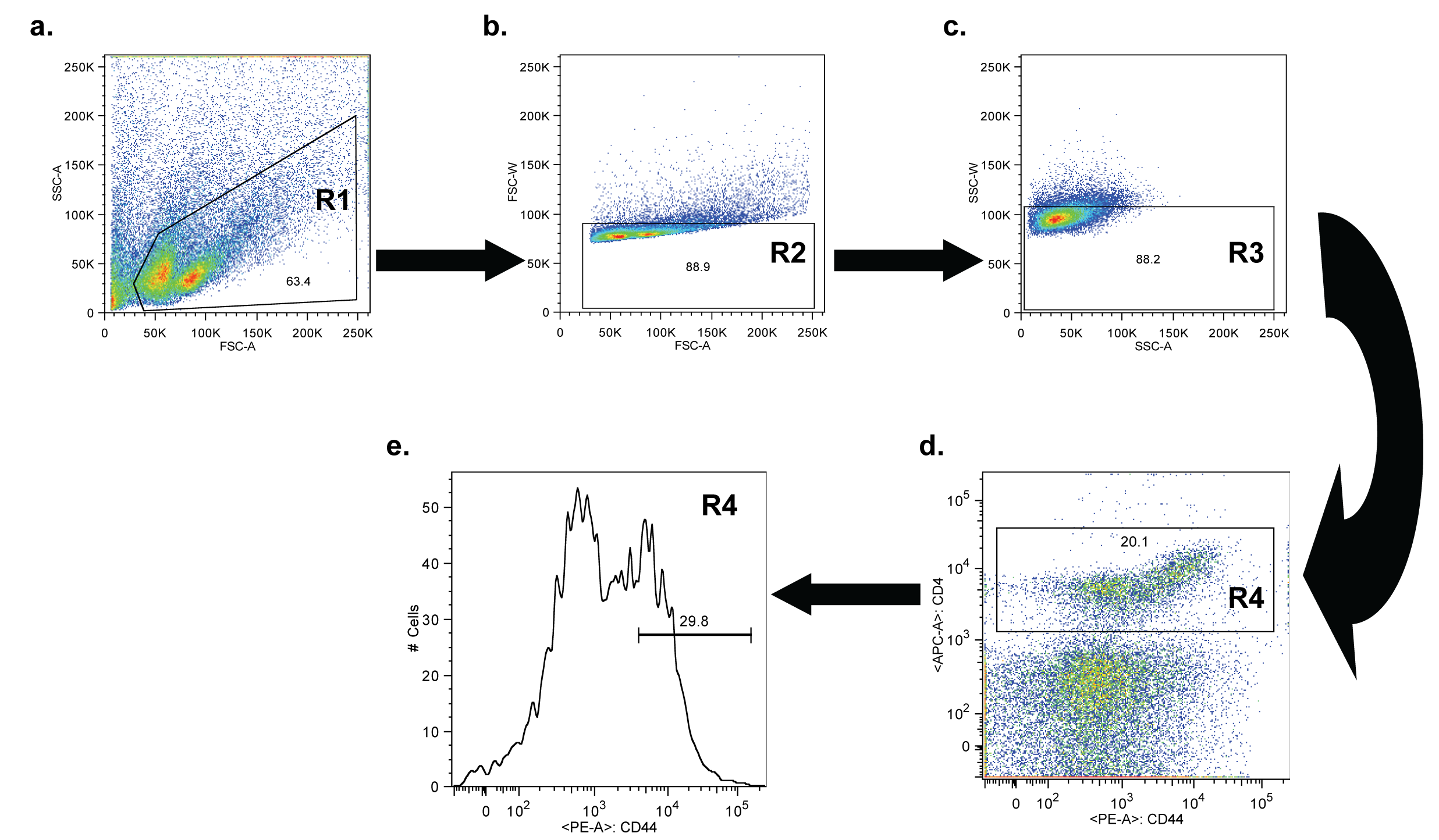

Supplement: Figure S1 — Flow cytometric cell-sorting strategy for isolation of CD4+CD44high cells. Islet-infiltrating cells were co-stained with fluorescent antibodies specific for CD4 and CD44. The figure shows representative FACS profiles of one 10-week-old NOD mouse and the strategy used for sorting. Regions R1 (panel a), R2 (panel b), and R3 (panel c) indicate selection of lymphocytes and exclusion of doublets. Region R4 in panel d identifies CD4+ cells. Panel e shows the expression of CD44 on gated CD4+ T cells (R4). The marker in panel e shows the region used to sort memory CD4+CD44high cells, which were defined as CD4+ T cells expressing the highest level of CD44 (in this case 29.8% of CD4+CD44high cells). (TIF) [file pone.0076546.s001.tif]

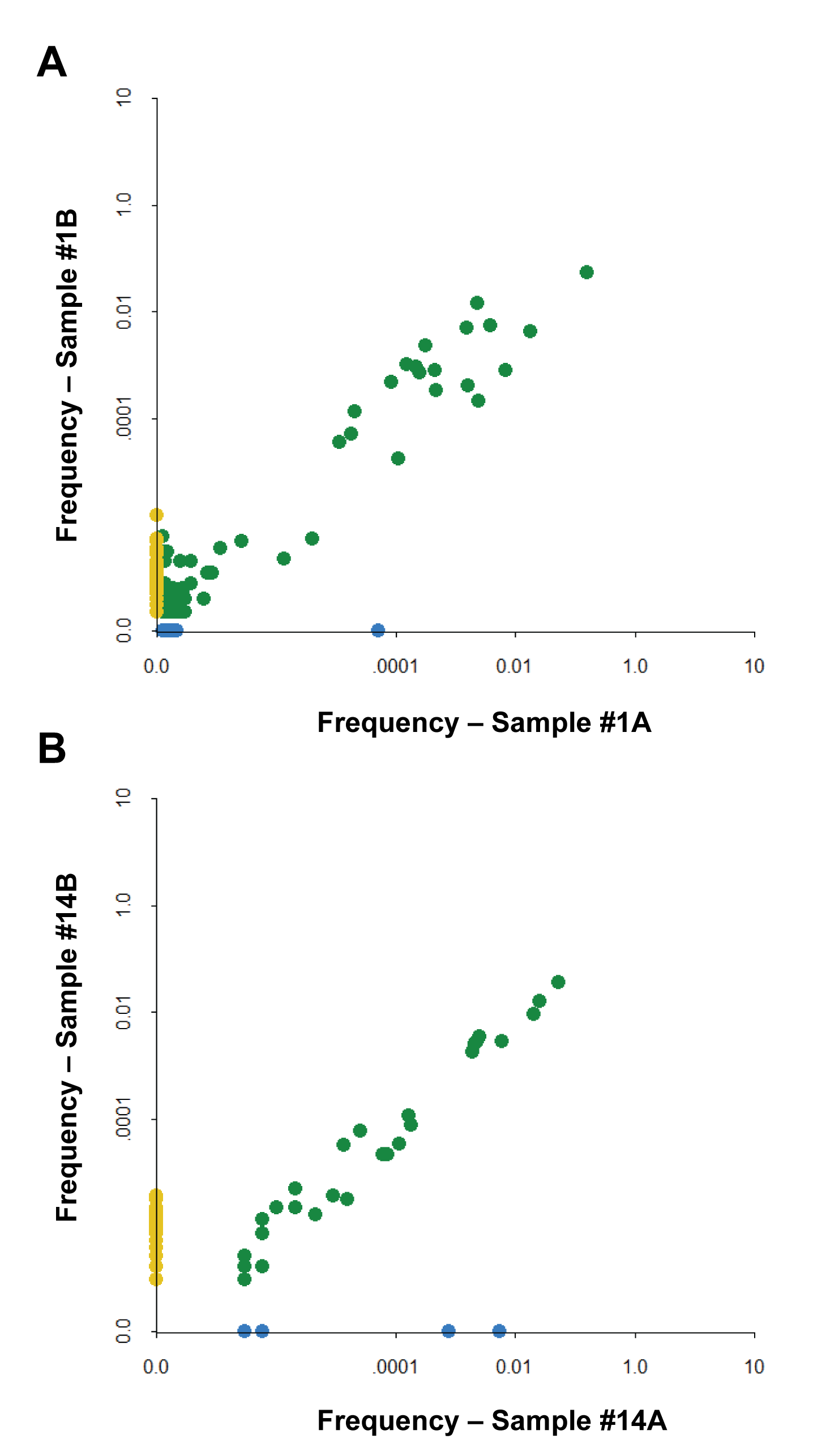

Supplement: Figure S2 — Validation of the sequencing protocol. Sequence copy counts of TCRβ CDR3 regions obtained by sequencing the same cDNA sample from prediabetic mouse #1 (A) and diabetic mouse #14 (B) in two separate PCR reactions. Each point in the log–log scatter plot represents a unique clone. Points in green were found in both reactions and those in blue or yellow were found in one PCR reaction. (TIF) [file pone.0076546.s002.tif]

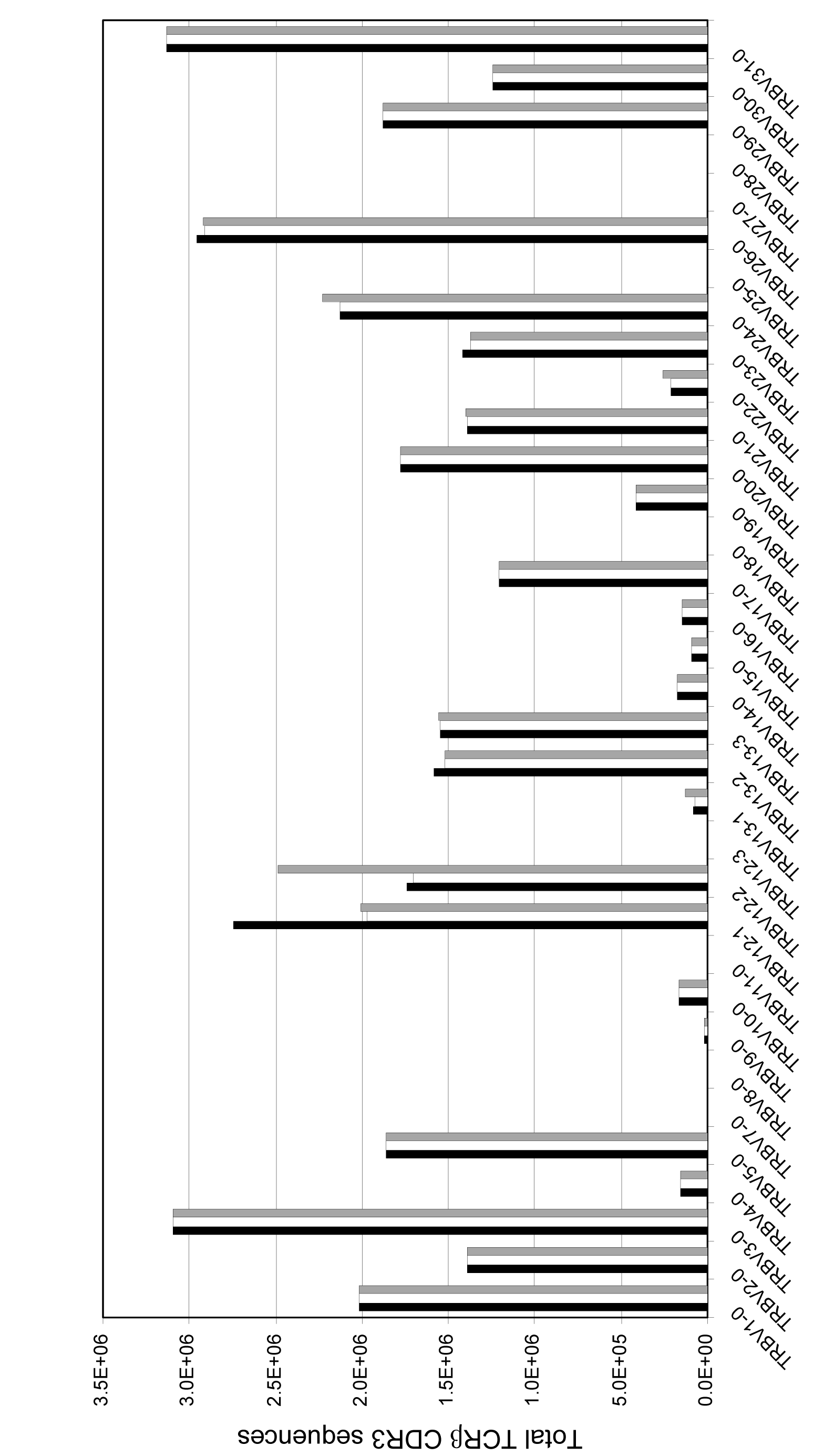

Supplement: Figure S3 — PCR bias assessment. Each Vβ primer was PCR amplified and sequenced against all Jβ primers. After processing, all TRBV gene sequences were mapped and recorded. The graph shows the combined data set for all TRBV genes. The bars correspond to the number of TRBV segments correctly assigned (white), the number miss-assigned to a particular TRBV gene (black) and the number of sequences incorrectly assigned from a particular TRBV gene (grey). (TIF) [file pone.0076546.s003.tif]

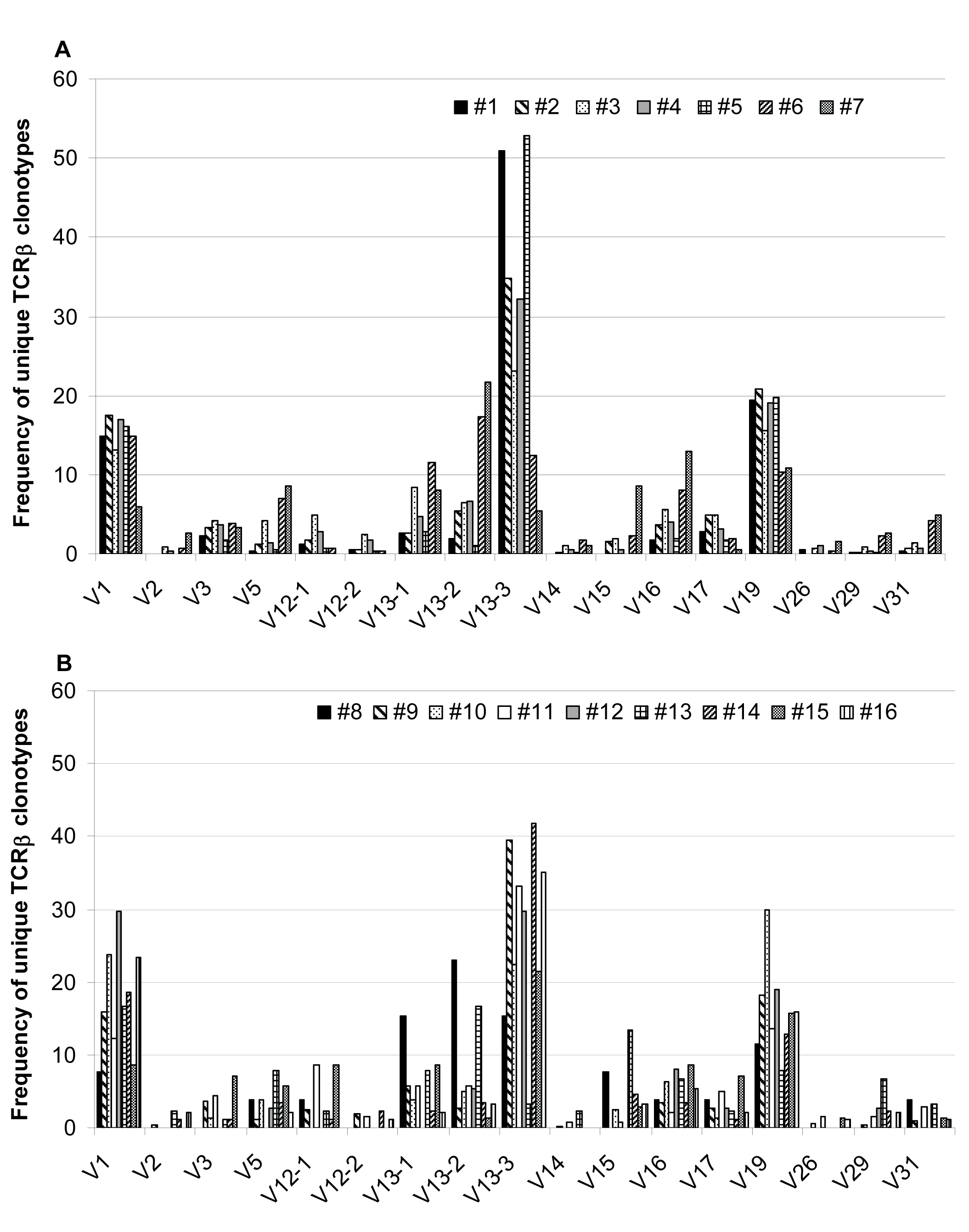

Supplement: Figure S4 — TRBV gene usage by islet-infiltrating CD4+CD44high clonotypes from individual NOD mice. The frequency of TRBV gene usage in each of the prediabetic (A) and newly diabetic (B) mice. (TIF) [file pone.0076546.s004.tif]
