# Supplementary material for: High-Throughput Sequencing of Islet-Infiltrating Memory CD4+ T Cells Reveals a Similar Pattern of TCR Vβ Usage in Prediabetic and Diabetic NOD Mice
Source: PLoS One. 2013 Oct 17;8(10):e76546. doi: 10.1371/journal.pone.0076546 (PMC3798422; doi:10.1371/journal.pone.0076546)
Supplement: Table S1 — Frequency of TRBV gene segment usage by individual mice. The frequency of TRBV gene segment usage by total, high-frequency, and rare islet-infiltrating CD4+CD44high clonotypes. (DOC) [file pone.0076546.s005.doc]

| **Table S1. Frequency of TRBV gene usage by total CD4^+^CD44^high^ clonotypes from individual mice** | | | | | | | | | | | | | | | | |
| --- | --- | --- | --- | --- | --- | --- | --- | --- | --- | --- | --- | --- | --- | --- | --- | --- |
|  | **Prediabetic NOD mice** | | | | | | | **Diabetic NOD mice** | | | | | | | | |
| **TRBV** | **#1** | **#2** | **#3** | **#4** | **#5** | **#6** | **#7** | **#8** | **#9** | **#10** | **#11** | **#12** | **#13** | **#14** | **#15** | **#16** |
| **# clones** | **1989** | **388** | **798** | **7692** | **2049** | **2822** | **185** | **26** | **849** | **80** | **139** | **37** | **90** | **86** | **70** | **94** |
| **V1** | 14.9 | 17.5 | 13.2 | 17.0 | 16.1 | 14.9 | 6.0 | 7.7 | 15.9 | 23.8 | 12.2 | 29.7 | 16.7 | 18.6 | 8.6 | 23.4 |
| **V2** | 0.1 |  | 0.9 | 0.4 | 0.1 | 0.8 | 2.7 |  | 0.5 |  |  |  | 2.2 | 1.2 |  | 2.1 |
| **V3** | 2.2 | 3.4 | 4.1 | 3.7 | 1.8 | 3.8 | 3.2 |  | 3.7 | 1.3 | 4.3 |  | 1.1 | 1.2 | 7.1 |  |
| **V4** |  |  |  | 0.01 |  | 0.04 |  |  |  |  |  |  |  |  |  |  |
| **V5** | 0.4 | 1.3 | 4.1 | 1.4 | 0.5 | 7.0 | 8.7 | 3.9 | 1.2 | 3.8 |  | 2.7 | 7.8 | 3.5 | 5.7 | 2.1 |
| **V12-1** | 1.3 | 1.8 | 4.9 | 2.7 | 0.6 | 0.6 |  | 3.9 | 2.5 |  | 8.6 |  | 2.2 | 1.2 | 8.6 |  |
| **V12-2** | 0.5 | 0.5 | 2.5 | 1.8 | 0.4 | 0.3 |  |  | 2.0 |  | 1.4 |  |  | 2.3 |  | 1.1 |
| **V13-1** | 2.6 | 2.6 | 8.4 | 4.7 | 2.8 | 11.5 | 8.1 | 15.4 | 5.8 | 3.8 | 5.8 |  | 7.8 | 2.3 | 8.6 | 2.1 |
| **V13-2** | 1.9 | 5.4 | 6.5 | 6.6 | 1.1 | 17.3 | 21.6 | 23.1 | 2.6 | 5.0 | 5.8 | 5.4 | 16.7 | 3.5 | 1.4 | 3.2 |
| **V13-3** | 50.9 | 34.8 | 23.1 | 32.2 | 52.8 | 12.5 | 5.4 | 15.4 | 39.5 | 22.5 | 33.1 | 29.7 | 3.3 | 41.9 | 21.4 | 35.1 |
| **V14** | 0.1 | 0.3 | 1.1 | 0.5 | 0.1 | 1.8 | 1.1 |  | 0.2 |  | 0.7 |  | 2.2 |  |  |  |
| **V15** | 0.1 | 1.6 | 1.9 | 0.6 |  | 2.2 | 8.7 | 7.7 |  | 2.5 | 0.7 |  | 13.3 | 4.7 | 2.9 | 3.2 |
| **V16** | 1.8 | 3.6 | 5.6 | 4.1 | 1.9 | 8.1 | 13.0 | 3.9 | 3.5 | 6.3 | 2.2 | 8.1 | 6.7 | 3.5 | 8.6 | 5.3 |
| **V17** | 2.8 | 4.9 | 4. 9 | 3.1 | 1.8 | 2.0 | 0.5 | 3.9 | 2.6 | 1.3 | 5.0 | 2.7 | 2.2 | 1.2 | 7.1 | 2.1 |
| **V19** | 19.4 | 20.9 | 15.5 | 19.0 | 19.7 | 10.3 | 10.8 | 11.5 | 18.1 | 30.0 | 13.7 | 18.9 | 7.8 | 12.8 | 15.7 | 16.0 |
| **V20** | 0.1 | 0.3 | 0.1 |  |  | 0.1 |  |  |  |  |  |  |  |  |  |  |
| **V21** |  | 0.3 |  | 0.1 |  |  |  |  |  |  | 0.7 |  |  |  |  |  |
| **V22** |  |  |  | 0.01 |  |  |  |  |  |  |  |  |  |  | 1.4 |  |
| **V23** |  |  | 0.1 | 0.1 |  | 0.04 |  |  |  |  |  |  |  |  |  |  |
| **V24** |  |  |  | 0.1 |  |  | 1.1 |  | 0.1 |  |  |  |  |  |  |  |
| **V26** | 0.5 |  | 0.8 | 1.0 | 0.1 | 0.4 | 1.6 |  | 0.6 |  | 1.4 |  |  |  | 1.4 | 1.1 |
| **V29** | 0.3 | 0.3 | 0.9 | 0.3 | 0.2 | 2.3 | 2.7 |  | 0.4 |  | 1.4 | 2.7 | 6.7 | 2.3 |  | 2.1 |
| **V30** |  |  |  |  | 0.1 | 0.1 |  |  |  |  |  |  |  |  |  |  |
| **V31** | 0.4 | 0.8 | 1.4 | 0.7 |  | 4.2 | 4.9 | 3.9 | 0.9 |  | 2.9 |  | 3.3 |  | 1.4 | 1.1 |
| **Frequency of TRBV gene usage by high-frequency CD4^+^CD44^high^ clonotypes from individual mice** | | | | | | | | | | | | | | | |  |
|  | **Prediabetic NOD mice** | | | | | | **Diabetic NOD mice** | | | | | | | | |  |
| **TRBV** | **#1** | **#2** | **#3** | **#4** | **#6** | **#7** | **#8** | **#9** | **#10** | **#11** | **#12** | **#13** | **#14** | **#15** | **#16** |  |
| **# clones** | **15** | **16** | **16** | **10** | **2** | **13** | **13** | **0** | **5** | **6** | **4** | **15** | **10** | **9** | **8** |  |
| **V1** | 26.7 | 25 | 50 | 30 |  | 30.8 | 6.3 |  | 20 | 50 | 25 | 60 | 40 |  | 12.5 |  |
| **V2** |  |  |  | 10 |  |  |  |  |  |  |  |  |  |  | 12.5 |  |
| **V3** | 6.7 |  |  |  |  |  |  |  |  |  |  |  |  | 11 |  |  |
| **V5** | 6.7 |  |  |  |  |  | 6.3 |  |  |  |  | 6.7 |  |  |  |  |
| **V12-1** | 6.7 |  |  |  |  |  |  |  |  |  |  |  |  |  |  |  |
| **V13-1** | 13.3 |  |  |  |  | 7.7 | 12.5 |  | 40 | 16.7 |  | 6.7 | 10 | 22 | 12.5 |  |
| **V13-2** |  | 12.5 |  |  |  | 7.7 | 25 |  |  | 16.7 | 25 |  |  |  |  |  |
| **V13-3** |  | 25 | 25 | 30 | 100 | 7.7 | 6.3 |  |  | 16.7 |  | 6.7 | 20 | 22 |  |  |
| **V15** |  |  |  | 30 |  |  | 6.3 |  |  |  |  | 6.7 |  | 11 | 25 |  |
| **V16** | 20 | 6.3 | 6.3 |  |  | 7.7 | 6.3 |  |  |  | 25 | 6.7 |  | 22 | 12.5 |  |
| **V17** |  | 6.3 |  |  |  |  |  |  | 20 |  |  |  |  | 11 | 12.5 |  |
| **V19** | 20 | 25 | 18.8 |  |  | 38.5 | 6.3 |  | 20 |  |  | 6.7 | 30 |  | 12.5 |  |
| **V29** |  |  |  |  |  |  |  |  |  |  | 25 |  |  |  |  |  |
| **V31** |  |  |  |  |  |  | 6.3 |  |  |  |  |  |  |  |  |  |

| **Frequency of TRBV gene usage by rare CD4^+^CD44^high^ clonotypes from individual mice** | | | | | | | | | | | | | | | | |
| --- | --- | --- | --- | --- | --- | --- | --- | --- | --- | --- | --- | --- | --- | --- | --- | --- |
|  | **Prediabetic NOD mice** | | | | | | | **Diabetic NOD mice** | | | | | | | | |
| **TRBV** | **#1** | **#2** | **#3** | **#4** | **#5** | **#6** | **#7** | **#8** | **#9** | **#10** | **#11** | **#12** | **#13** | **#14** | **#15** | **#16** |
| **# clones** | **1974** | **372** | **782** | **7682** | **2049** | **2820** | **172** | **13** | **849** | **75** | **133** | **34** | **75** | **76** | **61** | **85** |
| **V1** | 14.8 | 17.2 | 12.4 | 17.0 | 16.1 | 14.9 | 4.1 | 7.7 | 15.9 | 24.0 | 10.5 | 29.4 | 8.0 | 15.8 | 9.8 | 24.7 |
| **V2** | 0.1 |  | 0.9 | 0.4 | 0.05 | 0.8 | 2.9 |  | 0.5 |  |  |  | 2.7 | 1.3 |  | 1.2 |
| **V3** | 2.2 | 3.5 | 4.2 | 3.7 | 1.8 | 3.8 | 3.5 |  | 3.7 | 1.3 | 4.5 |  | 1.3 | 1.3 | 6.6 |  |
| **V4** |  |  |  | 0.01 |  | 0.04 |  |  |  |  |  |  |  |  |  |  |
| **V5** | 0.4 | 1.3 | 4.2 | 1.4 | 0.5 | 7.0 | 9.3 |  | 1.2 | 4.0 |  | 2.9 | 8.0 | 3.9 | 6.6 | 2.4 |
| **V12-1** | 1.3 | 1.9 | 5.0 | 2.7 | 0.6 | 0.6 |  | 7.7 | 2.5 |  | 9.0 |  | 2.7 | 1.3 | 9.8 |  |
| **V12-2** | 0.5 | 0.6 | 2.6 | 1.8 | 0.4 | 0.3 |  |  | 2.0 |  | 1.5 |  |  | 2.6 |  | 1.2 |
| **V13-1** | 2.5 | 2.7 | 8.6 | 4.7 | 2.8 | 11.5 | 8.1 | 15.4 | 5.8 | 1.3 | 5.3 |  | 8.0 | 1.3 | 6.6 | 1.2 |
| **V13-2** | 1.9 | 5.1 | 6.6 | 6.6 | 1.1 | 17.3 | 22.7 | 15.4 | 2.6 | 5.3 | 5.3 | 2.9 | 20.0 | 3.9 | 1.6 | 3.5 |
| **V13-3** | 51.3 | 35.2 | 23.0 | 32.2 | 52.8 | 12.4 | 5.2 | 23.1 | 39.5 | 24.0 | 33.8 | 32.4 | 2.7 | 44.7 | 21.3 | 38.8 |
| **V14** | 0.1 | 0.3 | 1.2 | 0.5 | 0.1 | 1.8 | 1.2 |  | 0.2 |  | 0.8 |  | 2.7 |  |  |  |
| **V15** | 0.1 | 1.6 | 1.9 | 0.5 |  | 2.2 | 9.3 | 7.7 |  | 2.7 | 0.8 |  | 14.7 | 5.3 | 1.6 | 1.2 |
| **V16** | 1.7 | 3.5 | 5.6 | 4.1 | 1.9 | 8.1 | 13.4 |  | 3.5 | 6.7 | 2.3 | 5.9 | 6.7 | 3.9 | 6.6 | 4.7 |
| **V17** | 2.8 | 4.8 | 5.0 | 3.1 | 1.8 | 2.0 | 0.6 | 7.7 | 2.6 |  | 5.3 | 2.9 | 2.7 | 1.3 | 6.6 | 1.2 |
| **V19** | 19.4 | 20.7 | 15.5 | 19.0 | 19.7 | 10.3 | 8.7 | 15.4 | 18.1 | 30.7 | 14.3 | 20.6 | 8.0 | 10.5 | 18.0 | 16.5 |
| **V20** | 0.1 | 0.3 | 0.1 |  |  | 0.1 |  |  |  |  |  |  |  |  |  |  |
| **V21** |  | 0.3 |  | 0.1 |  |  |  |  |  |  | 0.8 |  |  |  |  |  |
| **V22** |  |  |  | 0.01 |  |  |  |  |  |  |  |  |  |  | 1.6 |  |
| **V23** |  |  | 0.1 | 0.1 |  | 0.04 |  |  |  |  |  |  |  |  |  |  |
| **V24** |  |  |  | 0.1 |  |  | 1.2 |  | 0.1 |  |  |  |  |  |  |  |
| **V26** | 0.5 |  | 0.8 | 1.0 | 0.05 | 0.4 | 1.7 |  | 0.6 |  | 1.5 |  |  |  | 1.6 | 1.2 |
| **V29** | 0.3 | 0.3 | 0.9 | 0.3 | 0.2 | 2.3 | 2.9 |  | 0.4 |  | 1.5 | 2.9 | 8.0 | 2.6 |  | 1.2 |
| **V30** |  |  |  |  | 0.05 | 0.1 |  |  |  |  |  |  |  |  |  |  |
| **V31** | 0.4 | 0.8 | 1.4 | 0.7 |  | 4.2 | 5.2 |  | 0.9 |  | 3.0 |  | 4.0 |  | 1.6 | 1.2 |
