# Supplementary material for: High-Throughput Sequencing of Islet-Infiltrating Memory CD4+ T Cells Reveals a Similar Pattern of TCR Vβ Usage in Prediabetic and Diabetic NOD Mice
Source: PLoS One. 2013 Oct 17;8(10):e76546. doi: 10.1371/journal.pone.0076546 (PMC3798422; doi:10.1371/journal.pone.0076546)
Supplement: Table S2 — Islet-infiltrating CD4+CD44high clonotypes shared by at least one prediabetic and one diabetic mouse. 135 clonotypes common to both groups of mice were shared by at least one mouse from each group. (DOC) [file pone.0076546.s006.doc]

**Table S2. Islet-infiltrating CD4^+^CD44^high^ clonotypes present in prediabetic and diabetic NOD mice**

| CDR3 sequence^1^ | TRBV | TRBJ | CDR3 length |  | CDR3 sequence | TRBV | TRBJ | CDR3 length |
| --- | --- | --- | --- | --- | --- | --- | --- | --- |
| SADRANTEVFF^2^ | 1 | 1-1 | 11 |  | SLSYEQYF | 3 | 2-7 | 8 |
| SADNRVDTEVFF | 1 | 1-1 | 12 |  | SRQGNTGQLYF | 5 | 2-2 | 11 |
| SADGNSDYTF | 1 | 1-2 | 10 |  | SLDIYAEQFF | 12 | 2-1 | 10 |
| SGGQNSDYTF | 1 | 1-2 | 10 |  | SLEGNYAEQFF | 12 | 2-1 | 11 |
| SADRNSDYTF | 1 | 1-2 | 10 |  | SRDWGGAEQFF | 12 | 2-1 | 11 |
| SADRANSDYTF^2^ | 1 | 1-2 | 11 |  | SEGSDYTF | 13-1 | 1-2 | 8 |
| SADGTANSDYTF | 1 | 1-2 | 12 |  | SENSDYTF | 13-1 | 1-2 | 8 |
| SGDNSGNLYF | 1 | 1-3 | 10 |  | SDANSDYTF | 13-1 | 1-2 | 9 |
| SADRVSNERLFF | 1 | 1-4 | 12 |  | SDEGNSDYTF | 13-1 | 1-2 | 10 |
| SGTISNERLFF | 1 | 1-4 | 11 |  | SDQGNSDYTF | 13-1 | 1-2 | 10 |
| SAYNSLYF | 1 | 1-6 | 8 |  | SARSDSDYTF | 13-1 | 1-2 | 10 |
| SGSYNSLYF | 1 | 1-6 | 9 |  | SGGYAEQFF | 13-1 | 2-1 | 9 |
| SAGGNSLYF | 1 | 1-6 | 9 |  | SGQYAEQFF | 13-1 | 2-1 | 9 |
| SAGGYNSLYF^2^ | 1 | 1-6 | 10 |  | RTGANTGQLYF | 13-1 | 2-2 | 11 |
| SGQNSYNSLYF | 1 | 1-6 | 11 |  | SDGSSAETLYF | 13-1 | 2-3 | 11 |
| SAGAEQFF | 1 | 2-1 | 8 |  | SGTSQNTLYF | 13-1 | 2-4 | 10 |
| SADAEQFF | 1 | 2-1 | 8 |  | RDWGQNTLYF | 13-1 | 2-4 | 10 |
| SAGDYAEQFF | 1 | 2-1 | 10 |  | SDVGYEQYF | 13-1 | 2-7 | 9 |
| SGTGGVAEQFF | 1 | 2-1 | 11 |  | GDTANSDYTF | 13-2 | 1-2 | 10 |
| SAGTDYAEQFF | 1 | 2-1 | 11 |  | GGDRGWNSLYF | 13-2 | 1-6 | 11 |
| SADLGGYAEQFF | 1 | 2-1 | 12 |  | GDYAEQFF | 13-2 | 2-1 | 8 |
| SADRQNYAEQFF | 1 | 2-1 | 12 |  | GDAGVNYAEQFF | 13-2 | 2-1 | 12 |
| SATGDTGQLYF | 1 | 2-2 | 11 |  | GDGGAETLYF | 13-2 | 2-3 | 10 |
| SADLGGTGQLYF | 1 | 2-2 | 12 |  | GVQDTQYF | 13-2 | 2-5 | 8 |
| SGSQNTLYF^2^ | 1 | 2-4 | 9 |  | GGGDTQYF | 13-2 | 2-5 | 8 |
| SAGTGGNTLYF | 1 | 2-4 | 11 |  | GVQDTQYF | 13-2 | 2-5 | 8 |
| SAEGGQNTLYF | 1 | 2-4 | 11 |  | GDANSQDTQYF | 13-2 | 2-5 | 11 |
| SADKDTQYF | 1 | 2-5 | 9 |  | GDAWDEQYF | 13-2 | 2-7 | 9 |
| SAGLGGYEQYF | 1 | 2-7 | 11 |  | SAGDTEVFF | 13-3 | 1-1 | 9 |
| SAERDWGYEQYF | 1 | 2-7 | 12 |  | SPGQANTEVFF | 13-3 | 1-1 | 11 |
| SQEERGPSAETLYF | 2 | 2-3 | 14 |  | SRQNSDYTF | 13-3 | 1-2 | 9 |
| SQEGQGYEQYF | 2 | 2-7 | 11 |  | RDRANSDYTF | 13-3 | 1-2 | 10 |
|  |  |  |  |  |  |  |  |  |

^1^ CDR3β sequences shared by two or three mice.

^2^ Clonotypes shared by three mice.

| CDR3 sequence | TRBV | TRBJ | CDR3 length |  | CDR3 sequence | TRBV | TRBJ | CDR3 length |
| --- | --- | --- | --- | --- | --- | --- | --- | --- |
| SDRANSDYTF^2^ | 13-3 | 1-2 | 10 |  | SDWGSSQNTLYF^2^ | 13-3 | 2-4 | 12 |
| SDAGVNSDYTF | 13-3 | 1-2 | 11 |  | RDNQDTQYF | 13-3 | 2-5 | 9 |
| SEDRPNSDYTF | 13-3 | 1-2 | 11 |  | RGGQDTQYF | 13-3 | 2-5 | 9 |
| SDRDKNSDYTF | 13-3 | 1-2 | 11 |  | SDRDQDTQYF^2^ | 13-3 | 2-5 | 10 |
| SDAGGDSDYTF | 13-3 | 1-2 | 11 |  | SDGGGQDTQYF | 13-3 | 2-5 | 11 |
| SDRGSGNLYF | 13-3 | 1-3 | 10 |  | SPGGARDTQYF | 13-3 | 2-5 | 11 |
| SGTISNERLFF^2^ | 13-3 | 1-4 | 11 |  | SDLYEQYF | 13-3 | 2-7 | 8 |
| SESNSLYF | 13-3 | 1-6 | 8 |  | RTGGYEQYF | 13-3 | 2-7 | 9 |
| SGQNNSLYF | 13-3 | 1-6 | 9 |  | SDLGGYEQYF | 13-3 | 2-7 | 10 |
| SDSYNSLYF | 13-3 | 1-6 | 9 |  | SPGAEQFF^2^ | 15 | 2-1 | 8 |
| RGSYNSLYF | 13-3 | 1-6 | 9 |  | SLGDAEQFF | 15 | 2-1 | 9 |
| SDGQYNSLYF | 13-3 | 1-6 | 10 |  | SLKYEQYF | 15 | 2-7 | 8 |
| SDSSYNSLYF^2^ | 13-3 | 1-6 | 10 |  | SPGTNEQYF | 15 | 2-7 | 9 |
| SGTVSYNSLYF | 13-3 | 1-6 | 11 |  | SLDRYEQYF | 15 | 2-7 | 9 |
| SDRDRGYNSLYF | 13-3 | 1-6 | 12 |  | SLDSSYEQYF^2^ | 15 | 2-7 | 10 |
| SDLGNYAEQFF^2^ | 13-3 | 2-1 | 11 |  | SLGTGGAAEVFF | 16 | 1-1 | 12 |
| SGTGGYAEQFF^2^ | 13-3 | 2-1 | 11 |  | SLVGGNYAEQFF^2^ | 16 | 2-1 | 12 |
| SELGDYAEQFF^2^ | 13-3 | 2-1 | 11 |  | SIGGNSDYTF | 19 | 1-2 | 10 |
| SDTGGYAEQFF^2^ | 13-3 | 2-1 | 11 |  | SSGTTNSDYTF | 19 | 1-2 | 11 |
| SDAGGNYAEQFF | 13-3 | 2-1 | 12 |  | SRQGANSDYTF^2^ | 19 | 1-2 | 11 |
| SDWGGNYAEQFF | 13-3 | 2-1 | 12 |  | SRRNNNQAPLF | 19 | 1-5*1 | 11 |
| SGLGGNYAEQFF | 13-3 | 2-1 | 12 |  | RTGVNSLYF | 19 | 1-6 | 9 |
| SGRTGGYAEQFF | 13-3 | 2-1 | 12 |  | SIGNSYNSLYF | 19 | 1-6 | 11 |
| SDGAEQFF | 13-3 | 2-1 | 8 |  | SIYAEQFF | 19 | 2-1 | 8 |
| SDRTGYAEQFF | 13-3 | 2-1 | 11 |  | STGGYAEQFF | 19 | 2-1 | 10 |
| RDWGNYAEQFF | 13-3 | 2-1 | 11 |  | SPGQNYAEQFF | 19 | 2-1 | 11 |
| SGDRDYAEQFF | 13-3 | 2-1 | 11 |  | SIWGGDAEQFF | 19 | 2-1 | 11 |
| SANTGQLYF | 13-3 | 2-2 | 9 |  | SRTGGNYAEQFF | 19 | 2-1 | 12 |
| SGTGGYTGQLYF | 13-3 | 2-2 | 12 |  | SPGLGGRAEQFF | 19 | 2-1 | 12 |
| SDRASAETLYF | 13-3 | 2-3 | 11 |  | SIWGGNTGQLYF | 19 | 2-2 | 12 |
| SETGGAETLYF | 13-3 | 2-3 | 11 |  | SIDGGRAETLYF^2^ | 19 | 2-3 | 12 |
| SRQGASAETLYF | 13-3 | 2-3 | 12 |  | SISQNTLYF^2^ | 19 | 2-4 | 9 |
| SEGTGSAETLYF | 13-3 | 2-3 | 12 |  | RPWGNQDTQYF | 19 | 2-5 | 11 |
| SDWGGASAETLYF^2^ | 13-3 | 2-3 | 13 |  | SISGGYEQYF | 19 | 2-7 | 10 |
| SDWENTLYF | 13-3 | 2-4 | 9 |  | SIGLGVSYEQYF^2^ | 19 | 2-7 | 12 |
| SDRGESQNTLYF | 13-3 | 2-4 | 12 |  | SLGDRGAEQFF | 31 | 2-1 | 11 |
